# A Versatile Vector Toolkit for Functional Analysis of Rice Genes

**DOI:** 10.1186/s12284-018-0220-7

**Published:** 2018-04-20

**Authors:** Feng He, Fan Zhang, Wenxian Sun, Yuese Ning, Guo-Liang Wang

**Affiliations:** 1 State Key Laboratory for Biology of Plant Diseases and Insect Pests, Institute of Plant Protection, Chinese Academy of Agricultural Sciences, Beijing, 100193 China; 2 College of Plant Protection, China Agricultural University, Beijing, 100193 China; 3 Department of Plant Pathology, The Ohio State University, Columbus, OH 43210 USA

**Keywords:** Vector toolkit, Transient expression, Binary vector, CRISPR/Cas9, *Oryza sativa*

## Abstract

**Background:**

Rice (*Oryza sativa*) is the main food for half of the world’s population, and is considered the model for molecular biology studies of monocotyledon species. Although the rice genome was completely sequenced about 15 years ago, the function of most rice genes is still unknown.

**Results:**

In this study, we developed a vector toolkit that contains 42 vectors for transient expression studies in rice protoplasts and stable expression analysis in transgenic rice. These vectors have been successfully used to study protein subcellular localization, protein-protein interaction, gene overexpression, and the CRISPR/Cas9-mediated gene editing. A novel feature of these vectors is that they contain a universal multiple cloning site, which enables more than 99% of the rice coding sequences to be conveniently transferred between vectors.

**Conclusions:**

The versatile vectors represent a highly efficient and high-throughput toolkit for functional analysis of rice genes.

**Electronic supplementary material:**

The online version of this article (10.1186/s12284-018-0220-7) contains supplementary material, which is available to authorized users.

## Background

Rice (*Oryza sativa*) is one of the most important food crops, as well as a model plant for studies of agronomic traits. The RICE2020 project was proposed by rice researchers in 2008 to characterize the functions of all rice genes (~ 32,000) by the year 2020 (Zhang et al. 2008). In the past 10 years, significant progress has been made with the availability of several gene-indexed rice mutant resources (Jung et al. 2008; Krishnan et al. 2009; Wang et al. 2013b), the development of large-scale omics profiles (Han et al. 2017; Lakshmanan et al. 2016; Wang et al. 2015), as well as advances in forward and reverse genetic technologies (Jiang et al. 2013; Schneeberger and Weigel 2011). To date, however, the functions of only a small proportion of rice genes have been reported. Elucidating the biological functions of the remaining genes is a daunting task for the rice research community (Li et al. 2018).

Vectors for gene ectopic expression, silencing, knock-out, promoter activity assay, protein subcellular localization, protein-protein interaction, and enzyme activity analysis are essential tools for gene characterization (Prelich 2012). The classic binary pCAMBIA vectors have been widely used for rice transformation in the past 20 years (Komori et al. 2007). Many specialized vectors for particular purposes have been derived from the pCAMBIA vectors, including the protein subcellular localization vector pGDG/pGDR (Goodin et al. 2002), the transposon Ac-Ds tagging vector pSQ5 (Qu et al. 2008), and the gene editing vectors such as the clustered regularly interspaced short palindromic repeats (CRISPR)/Cas9 system (Jiang et al. 2013; Xing et al. 2014; Zhou et al. 2014). Other vectors, such as pGreenII cloning vectors and pSAT vectors, have also been used for localization and for transient and stable expression of genes in plants (Hellens et al. 2005; Tzfira et al. 2005). In addition to the traditional restriction enzyme cloning strategy, the T-A cloning system was developed for gene cloning when appropriate restriction sites are lacking in the vector (Chen et al. 2009; Wang et al. 2013a). The T-A cloning system, however, is inconvenient for shuttling DNA fragments from one vector to another. At the same time, the cloning efficiency of the T-A cloning system is greatly reduced when the insert size is increased (Wang et al. 2013a). The Gateway system has also been widely applied in plants for over 15 years (Curtis and Grossniklaus 2003; Earley et al. 2006; Nakagawa et al. 2007). For example, the widely used rice RNA interference (RNAi) vector pANDA is based on the Gateway LR recombination (Miki and Shimamoto 2004; Wang et al. 2016). However, Gateway cloning requires an entry vector that matches with a full set of destination vectors, and the clonase used for the recombination reaction is expensive.

Because of the limitations of the TA and Gateway cloning systems, most laboratories still use classic restriction enzyme cloning. Unfortunately, multiple cloning sites (MCS) in the vectors that are suitable for the cloning of most rice genes are unavailable. To facilitate the transfer of rice fragments between vectors, we analyzed the restriction enzyme recognition sites in the rice coding sequences (CDSs) based on the MSU RGAP database Version7 (http://rice.plantbiology.msu.edu/), and we then synthesized a powerful universal MCS suitable for cloning of more than 99% of rice CDSs. At the same time, we optimized the individual components to generate small vectors with high efficiency. A total of 42 vectors were constructed for different applications, including gene transient expression, localization, Co-IP (co-immunoprecipitation), BiFC (bimolecular fluorescence complementation); as well as rice transgenic stable expression for gene editing, tissue or cell localization detection, promoter activity and gene complementation. We confirmed the utilities of these vectors with several assays in rice protoplasts, and stably transformed lines. Because the new vector toolkit is highly efficient, cost-effective, and easy to use, it will accelerate functional studies of genes in rice and other monocots.

## Results

### Construction of Vectors for Gene Functional Analysis

**Fig. 1:**
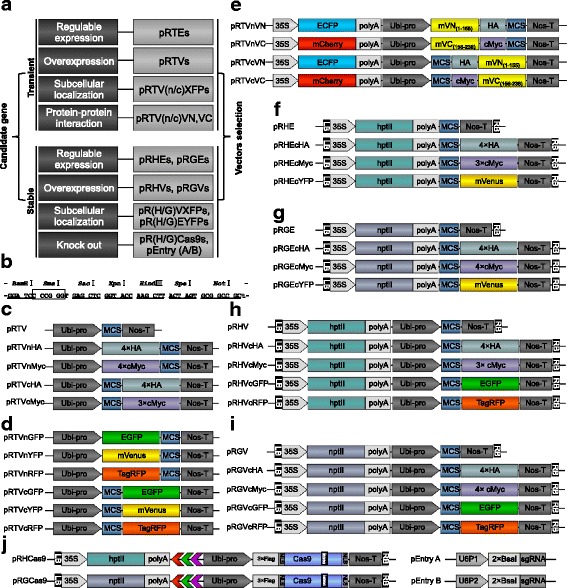
Schematic diagrams of the vectors. **a** Type and naming system of the 42 vectors. **b** The sequence and restriction enzymes in the multiple cloning site (MCS). **c**-**e** Vectors for rice transient overexpression. **c** pRTVs vectors with or without multiple N-/C- terminal HA tags or cMyc tags. **d** pRTVs vectors with N-/C- terminal fusion EGFP, mVenus, or TagRFP for subcellular localization in protoplasts. **e** Vectors for BiFC assays. mVN_(1–155)_ and mVC_(156–238)_ are the N and C terminal of mVenus, respectively. **f**-**j** Binary vectors for *Agrobacterium*-mediated transformation experiments. **f** and **g**, Vectors without the Ubi promoter for rice stable expression with hygromycin or G418 selection marker, respectively. **h** and **i**, Vectors for rice stable overexpression with hygromycin or G418 selection marker, respectively. **j** CRISPR/Cas9 vectors with hygromycin or G418 selection marker. *hptII*, the hygromycin selection marker gene; *nptII*, the G418 or kanamycin selection marker gene; LB, T-DNA left border; RB, T-DNA right border; NLS, nuclear localization signal; intron, second intron (IV2) of the potato gene *ST-LS1*

To construct an efficient vector system (Fig. 1a), we analyzed the efficiency of different expression elements in rice. Compared to the widely used cauliflower mosaic virus 35S promoter in dicots, the maize (*Zea mays*) ubiquitin-1 (Ubi) promoter is more efficient in monocots (Chen et al. 2009). However, the Ubi promoter contains many frequently used restriction sites, such as *Bgl*II, *Eco*RI, *Sal*I, *Xba*I, and *Xho*I (Additional file 1: Figure S1). We therefore systematically analyzed the restriction map of rice CDSs and selected seven abundant restriction sites that are not present in the Ubi promoter; at least two of the sites are absent in 99.55% of the rice CDSs (Table 1). As a consequence, these restriction sites can be used to clone 99.55% of the rice genes into these vectors using the classic double digestion-ligation strategy. We then synthesized these seven restriction sites in the order of *Bam*HI-*Sma*I-*Sac*I-*Kpn*I-*Hind*III-*Spe*I-*Not*I to form a universal MCS (Fig. 1b). To reduce the vector size, we used PCR to amplify the precise sequence of each element, and we then assembled the high copy number pUC replicon, kanamycin resistance gene, Ubi promoter, TagRFP, MCS, and Nos terminator (NosT). This generated the first vector, named pRTVnRFP, which is an abbreviation of the plasmid for rice transient overexpression with the N-terminal TagRFP tag. This vector contains only 4837 bp, which is suitable for gene expression and subcellular localization analysis in rice protoplasts (Fig. 1d).

**Table 1:** The cloning compatibility of the synthesized MCS for rice CDSs

CDSs	Number	Percentage	Total coverage
Containing 5 cut sites	1403	2.51	
Containing 6 cut sites	245	0.44	
Containing 7 cut sites	7	0.01	
Others (< 5 cut sites)	54,146	97.03	
Total	55,801	100	99.55%

Rice CDSs refer to the MSU RGAP database Version7 (http://rice.plantbiology.msu.edu/). Compatible genes should contain ≤5 restriction enzyme recognition sites of the MCS in double digestion-ligation cloning

We subsequently constructed a series of transient expression vectors, including the overexpression vectors with or without the N-/C-terminal epitope tag HA or cMyc (Fig. 1c), the fluorescent fusion expression vectors with the EGFP, mVenus or TagRFP tag (Fig. 1d), as well as the BiFC vectors with the mVenus (mVN_(1–155)_ and mVC_(156–238)_) halves (Fig. 1e). At the same time, we generated two vectors without the Ubi promoter for regulable expression (e.g., for application in promoter activity analysis and native expression and inducible expression assays) with or without the C-terminal mVenus (Additional file 1: Table S1).

Next, we constructed a set of binary transformation vectors using the transient expression vectors as backbones. The following were added to the transient expression vectors to make 21 transformation vectors: T-DNA border LB and RB, selection marker gene *HptII* (for hygromycin) or *NptII* (for G418/kanamycin) under the control of the 35S promoter, and the sequence ori-REP-STA from pVS1 for plasmid stabilization and replication in *A. tumefaciens*. Among the 21 transformation vectors, eight (four pRHEs and four pRGEs) did not contain the Ubi promoter and were suitable for gene regulable expression (Fig. 1f, g), and 13 vectors (8 pRHVs and 5 pRGVs) for gene overexpression (Fig. 1h, i, Additional file 1: Table S1). These vectors were classified and named according to their selection markers (with H standing for hygromycin and G standing for G418/kanamycin) and promoters (with E standing for regulable expression without the Ubi promoter, and V standing for overexpression with the Ubi promoter). Two CRISPR vectors, pRHCas9 and pRGCas9, were constructed for Cas9-mediated rice genome editing (Fig. 1j). The genome editing tool suites contain two additional compatible entry vectors, pEntry A and pEntry B, for packing the sgRNA cassette (Fig. 1j). These binary vectors can be used for almost all rice transgenic experiments.

### Applications for Protein Localization in Rice Protoplasts

To investigate the localization or co-localization of proteins in rice protoplasts, we constructed three kinds of N-/C-terminal fluorescent protein tags driven by the Ubi promoter, and we generated six overexpression vectors (pRTVnGFP, pRTVcGFP, pRTVnYFP, pRTVcYFP, pRTVnRFP, and pRTVcRFP) (Fig. 1d), as well as one vector (pRTEcYFP) that did not have the Ubi promoter but did have one of the brightest fluorescent tags, mVenus (Shaner et al. 2007) (Additional file 1: Table S1).

**Fig. 2:**
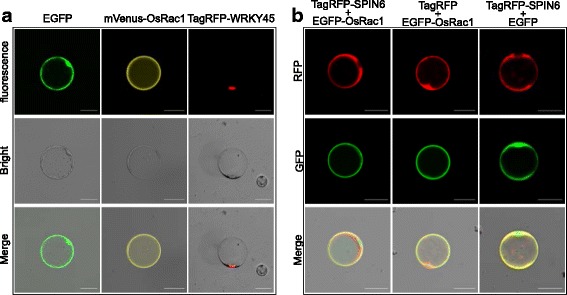
Applications for protein localization in rice protoplasts. **a** Subcellular localization assay. Plasmids pRTVnGFP, pRTVnYFP-OsRac1, and pRTVnRFP-WRKY45 were used for subcellular localization assays. Bar = 20 μm. **b** Co-localization assay. Plasmids pRTVnGFP-OsRac1, pRTVnRFP-SPIN6, and the corresponding empty vectors were used for co-localization assays. Bar = 20 μm

To confirm the utility of these vectors in protein subcellular localization, we tested pRTVnGFP, pRTVnYFP, and pRTVnRFP with the representative rice proteins OsRac1, WRKY45, and SPIN6 in localization assays. OsRac1, a member of Rac/Rop small GTPases and a key member of the rice defensome, was previously shown to localize in the plasma membrane (Akamatsu et al. 2013; Ono et al. 2001). The results showed that strong yellow fluorescence signals occurred on the protoplast membrane when transfected in rice protoplasts with pRTVnYFP-OsRac1 (Fig. 2a, middle column). Similarly, rice protoplasts transfected with pRTVnRFP-WRKY45, which contained the transcriptional factor WRKY45, a positive regulator of rice defense signaling (Shimono et al. 2007), showed specific red fluorescence signals in the nuclear region (Fig. 2a, right column). In contrast, the empty vector pRTVnGFP produced whole-cell localized GFP signals (Fig. 2a, left column).

These vectors were also used for the co-localization assay of SPIN6 and OsRac1. SPIN6 is a Rho GTPase-activating protein (RhoGAP) involved in inactivating OsRac1 and suppressing rice innate immunity (Liu et al. 2015). In the co-localization assay, we co-transfected rice protoplasts with plasmids pRTVnRFP-SPIN6 and pRTVnGFP-OsRac1. Compared with the EGFP and TagRFP controls whose signals were distributed throughout the cell, the red fluorescence signals of TagRFP-SPIN6 were mainly detected in the cytoplasm and plasma membrane, while the green fluorescence signals of EGFP-OsRac1 were detected in the plasma membrane, the signals of the two proteins merged and formed a yellow signal on the membrane (Fig. 2b). These experiments demonstrated that our vectors are suitable for protein localization and co-localization assays in rice protoplasts.

### Applications in Protein Transient Expression and Protein-Protein Interaction Assays

**Fig. 3:**
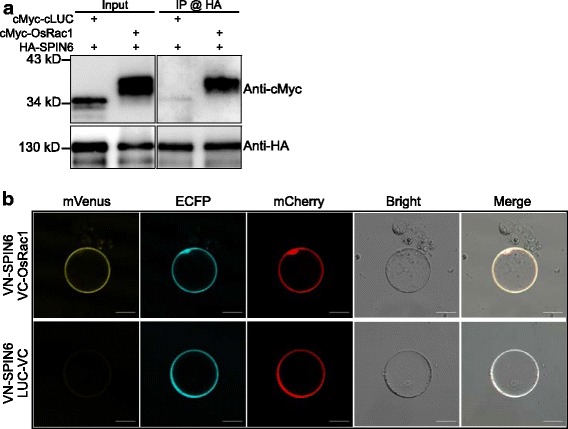
Applications in protein transient expression and protein-protein interactions. **a** Application for protein transient expression and Co-IP assay. Plasmids pRTVnHA-SPIN6, pRTVnMyc-OsRac1, and control pRTVnMyc-cLUC were used to express HA- or cMyc-tagged protein. cLUC_(417–568)_, C terminal of luciferase. **b** Application for BiFC assay. BiFC plasmids pRTVnVN-SPIN6, pRTVnVC-OsRac1, and control pRTVcVC-LUC were used for the interaction assay between SPIN6 and OsRac1. LUC, luciferase. Bar = 20 μm

Analysis of protein expression and protein-protein interaction is essential for understanding the molecular and biochemical functions of candidate genes (Hakes et al. 2008). Co-IP and BiFC assays are widely used for analysis of protein-protein interactions in vivo (Berggard et al. 2007; Liu et al. 2017; Wang et al. 2016). In this study, we constructed four vectors with multiple HA or cMyc epitope tags at their N-/C-terminals under the control of the Ubi promoter (pRTVnHA, pRTVnMyc, pRTVcHA, and pRTVcMyc) to facilitate immunoblotting, affinity purification, and Co-IP assay (Fig. 1c). To confirm the efficacy of our vectors for assessing protein expression and protein-protein interactions, we first carried out a Co-IP assay in rice protoplasts using these vectors with SPIN6 and OsRac1. The DNA fragments of the two genes were cloned into the pRTVnHA and pRTVnMyc vectors, respectively. We co-transfected rice protoplasts with the two plasmids and isolated total protein for western blot analysis. We detected the ~ 130-kDa HA-SPIN6 and ~ 40-kDa cMyc-OsRac1 proteins, as well as the control cMyc-cLUC (The C terminal 417–568 of luciferase) in the input (Fig. 3a). Moreover, the Co-IP result showed SPIN6 specifically binds to OsRac1 but not to cLUC (Fig. 3a), confirming the in vivo interaction between SPIN6 and OsRac1 as previously reported (Liu et al. 2015).

For BiFC assays, the two mutation sites V150A and I152L in the N-terminal of mVenus (mVN_(1–155)_) were introduced into our BiFC system to increase the signal-to-noise ratio (Kodama and Hu 2010; Nakagawa et al. 2011). Since the split-gene fragments in both plasmids are required to be expressed in the same cell in BiFC assay, we added the marker genes ECFP and mCherry into the VN (pRTVnVN and pRTVcVN) and VC (pRTVnVC and pRTVcVC) vectors, respectively, so that plasmid transfection and gene expression efficiency in the transfected cells can be assessed. We also added a fusion tag HA or cMyc to facilitate protein immunoblotting detection in the BiFC assay (Fig. 1e). We used these vectors to analyze the SPIN6 and OsRac1 interaction. *SPIN6* and *OsRac1* DNA fragments were fused with the split mVenus N-terminal and C-terminal in the vectors of pRTVnVN and pRTVnVC, respectively. After co-transfection, we detected clear yellow fluorescence signals on the plasma membrane of the transfected protoplasts, indicating the formation of reconstituted mVenus through the SPIN6-OsRac1 interaction (Fig. 3b). The ECFP and mCherry fluorescence in the control, in contrast, indicated successful transfection and expression of the two plasmids but did not indicate reconstitution of mVenus, which demonstrated the specific interaction between SPIN6 and OsRac1 (Fig. 3b). Together, these results suggested that our vectors are adequate for protein expression and protein-protein interaction assays in rice protoplasts.

### Applications for Generating Stable Transgenic Rice

**Fig. 4:**
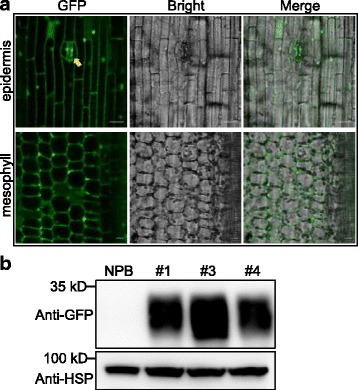
Application of overexpression vectors in generating GFP transgenic rice. **a** GFP signals in transgenic line #1 tissues. The arrow indicates the stoma. Vector pRHVnGFP was used for generating transgenic rice plants by *Agrobacterium*-mediated transformation. Bar = 10 μm. **b** Western blot detection of the GFP protein in transgenic lines. Three independent transgenic lines and the wild type were used for western blot detection. The HSP (heat shock protein) indicates the loading amount of each sample

We generated a series of vectors for stable expression in rice with hygromycin or G418/kanamycin selection markers. Epitope tags HA and cMyc, as well as fluorescent protein tags EGFP, TagRFP, and mVneus can be used for protein detection in transgenic plants. To check whether these vectors work well in rice, we selected the empty vector pRHVnGFP for *Agrobacterium*-mediated rice transformation. We obtained more than 20 transgenic plants in the Nipponbare (NPB) background. Confocal microscopy revealed strong, specific, green fluorescence signals in the epidermis, stomata guard cells, and mesophyll cells of the hygromycin-positive transgenic line #1 (Fig. 4a). We then confirmed the EGFP protein accumulation by western blot. We checked three independent hygromycin-positive lines (#1, #3, and #4). A strong, specific GFP band was detected in the three lines by western blot analysis compared to the wild type, NPB (Fig. 4b). The results indicated that these binary vectors worked properly in rice, and that they can be used in rice transgenic experiments, including gene overexpression assays, tissue or cell localization assays, promoter activity assays, and complementation assays.

### Applications of CRISPR/Cas9 Vectors for Generating Rice Mutants

**Fig. 5:**
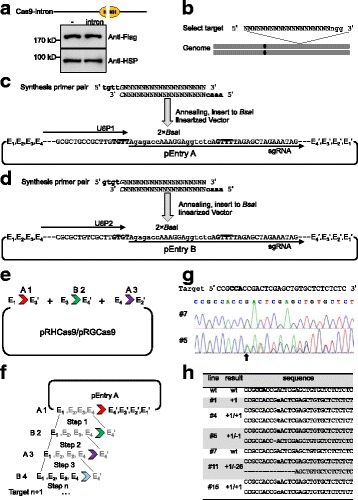
Diagram of how CRISPR/Cas9 vectors were constructed and used to edit the rice *IPA1* gene. **a** Western blot analysis of the *Cas9-intron* expression in rice protoplasts. HSP indicates the loading amount of each sample. **b** Target site selection for candidate genes in the rice genome. A 20-bp specific sequence followed by the PAM “NGG” structure is required. **c** and **d** Target cloning to the entry vectors. Synthesis of the primer pairs of the 20-bp specific target with the 4-bp adapters, and ligation with the *Bsa*I linearized pEntry A or pEntry B vector. **e** One-step ligation and **f** step-by-step ligation of multiple targets to pRHCas9/pRGCas9. Four pairs of isocaudamers, *Pst*I(E_1_)-*Nsi*I(E_1_’), *Xba*I(E_2_)-*Spe*I(E_2_’), *Bam*HI(E_3_)-*Bgl*I(E_3_’), and *Sal*I(E_4_)-*Xho*lI(E_4_’) are marked. The sgRNA cassettes with U6P1 and U6P2 in pEntry A and B should be used in turn. **g** Representative sequencing chromatogram of the CRISPR-*IPA1* transgenic lines. Line #7, wild-type genotype; line #5, mutant genotype. **h** Representative gene editing results in the CRISPR-*IPA1* transgenic lines

CRISPR/Cas9-mediated gene editing has been wildly used for functional studies of rice genes (Belhaj et al. 2015; Bi and Yang 2017; Ma et al. 2016). Most of the CRISPR/Cas9 vectors developed for rice are based on the pCAMBIA1300 vector (Ma et al. 2015; Piatek et al. 2015; Zhou et al. 2014). It is difficult to construct target sites in these CRISPR/Cas9 vectors through the Gateway cloning or the complicated Golden Gate Assembly strategy (Ma et al. 2015; Xie et al. 2015). In this study, we built a concise and accessible CRISPR/Cas9 system based on the pRHV and pRGV vectors. The two core parts, rice codon-optimized SpCas9 and sgRNA cassette, were cloned from a highly efficient genome editing system in a previous report (Zhou et al. 2014). To eliminate the toxicity of Cas9 in *Agrobacteria*, the second intron (*IV2*) of the *ST-LS1* gene (Thole et al. 2007) from potato was amplified and inserted into the Cas9 HNH nuclease domain. We confirmed the expression of the Cas9-intron in rice protoplasts by western blot analysis, and we detected the same size protein band with Cas9, indicating that the *IV2* intron was correctly spliced in rice cells (Fig. 5a). The construction of a new CRISPR/Cas9 vector is simple because only a *Bsa*I digestion is needed to insert the 20 bp gene-specific target fragments (target sequences should followed by a PAM sequence NGG, Fig. 5b) to pEntry A or pEntry B (Fig. 5c, d). Four pairs of isocaudamers were used for assembling the U6P-sgRNA cassette in the pEntry vectors (Additional file 1: Figure S2, Additional file 1: Figure S3). The two entry vectors should be used in turns when targeting multiple sites. Two strategies, one-step ligation and step-by-step ligation, could be used to assemble single or multiple targets (Fig. 5e, f). By using an one-step ligation strategy, we easily ligated three targets for *SPIN6* into pRHCas9.

To test our CRISPR/Cas9 vectors for generating rice mutants, we tried to edit the rice *IPA1* (Ideal Plant Architecture 1) gene, which is involved in the regulation of plant architecture and grain yield (Jiao et al. 2010). A previously reported CRIPSPR/Cas9 target sequence with complementary NGG PAM structure in *IPA1* gene (Li et al. 2016) was cloned into the pEntry A vector and then sub-cloned into the binary vector pRHCas9. Over 30 independent transgenic plants were generated through *Agrobacterium*-mediated rice transformation. We then randomly selected 20 plants for genotyping by PCR and sequencing. Among them, 16 (80%) showed gene editing at the target site of the *IPA1* gene (Fig. 5g), which was much higher than the editing rate (27.5%) of *IPA1* in the previous report (Li et al. 2016). Additionally, most of the edits appeared in a bi-allelic manner, and the most abundant editing pattern was one base pair insertion at 3 bp downstream of the complementary NGG PAM site (Fig. 5h). This result demonstrated that our CRISPR/Cas9 system has a high gene editing efficiency and is a useful tool for generating rice mutants.

## Discussion and Conclusions

To establish a highly efficient rice cell and plant experimental platform, we constructed a series of 41 vectors in this study. We added different modules from other systems, deleted most redundant sequences, and mutated the *Not*I recognition site between the connection region of pVS1 REP and STA from pCAMBIA1300. Furthermore, we tested these vectors with several genes in rice protoplasts and transgenic plants and demonstrated the usefulness of the system for the functional analysis of rice genes.

Except for the CRISPR/Cas9 suite, all of the vectors share the same MCS (Fig. 1b). The synthesized MCS with seven restriction sites is sufficient for cloning 99.55% of the rice genes. In other words, most of the rice genes can be easily cloned into our vectors through a double digestion-ligation strategy. In addition, the universal MCS is compatible with other commercial vectors, including prokaryotic expression with the MBP and GST fusion protein tag vectors pMalc2x and pGEX6p-1, and with the yeast-two-hybrid system pPC86 and pDBLeu vectors. The universal MCS is also helpful for fragment shuttling between vectors if necessary. The small size and high copy number of the vectors are ideal for efficient cloning, transformation, and plasmid preparation. For example, the pRTV vector for transient overexpression is only ~ 4.3 kb (Additional file 1: Figure S2). The plant overexpression vector pRHV is only 8.6 kb (Additional file 1: Figure S2), which is about 30% smaller than the 12-kb T-A cloning-based binary vector pCXUN (Chen et al. 2009). The remaining *Pst*I, *Eco*RV, *Not*I, *Asc*I, and *Pml*I recognition sites between modules may also be used for adding other components to the vectors (Additional file 1: Figure S2).

Functional analysis of candidate genes requires their transient expression in protoplasts and their stable expression in transgenic plants. Our vectors contain several epitope tags or fluorescent tags for protein expression analysis, including HA, cMyc, EGFP, mVenus, and TagRFP (Fig. 1 and Additional file 1: Table S1). BiFC assay is a popular method for determining protein-protein interactions and the interaction location in the cell. Researchers have improved the BiFC signal-to-noise ratio by selecting different fluorescent proteins, by split sites, and by creating point mutations (Kodama and Hu 2012). To increase BiFC reliability in rice protoplasts, we introduced the mutations V150A and I152L in the fluorescent protein Venus (Kodama and Hu 2010; Nakagawa et al. 2011) into the pRTVnVN and pRTVcVN vectors. The independently expressed ECFP and mCherry selection marker genes are used as controls to monitor the efficiency of construct transfection and gene expression in the treated cells in the BiFC system. The results from our subcellular localization, Co-IP, and BiFC assays are consistent with (or even better than) previous results (Akamatsu et al. 2013; Liu et al. 2015; Ono et al. 2001; Shimono et al. 2007) (Figs. 2, 3).

Hygromycin resistance is an effective selection marker in regenerating rice plants. An alternative selection marker such as G418/kanamycin resistance is required, however, when introducing another transgene or the CRISPR/Cas9 cassette into the existing hygromycin-resistant transgenic rice plants (Chakraborty et al. 2016; Park et al. 2016). In the 23 vectors suitable for rice transgenic experiments, 13 contain the *HptII* gene and 10 contain the *NptII* gene (Additional file 1: Table S1, Figure S2). To simplify target assembling, we have established the CRISPR/Cas9 system with four pairs of isocaudamers, and we have successfully used them to edit the rice *IPA1* gene, with an 80% gene editing efficiency. Therefore, our CRISPR/Cas9 vectors can be used to efficiently generate rice mutants.

Finally, some of the vectors developed in this research have been successfully used in our laboratory for several rice projects in the past few years. For example, we used the pRTVnHA vector to express HA-SPIN6 in protoplasts and to thereby suppress OsRac1 activity (Liu et al. 2015), and we used the pRTVnMyc vector to express cMyc-OsNPR1 in protoplasts and to thereby elucidate the OsCUL3a-OsNPR1 degradation mechanism (Liu et al. 2017). In conclusion, we have established a versatile vector toolkit for high efficiency and throughput gene functional analysis in rice. The vectors will be freely available to the community.

## Methods

### Plant Materials

Rice (*Oryza sativa* L. ssp. *japonica*) cultivar Nipponbare was used for protoplast isolation and transformation experiments. Rice plants were grown at 26–28 °C in greenhouses or growth chambers. For protoplast isolation, rice plants were kept in the dark for about 10 days.

### Plasmid Vector Construction

The construction of the vectors was based on PCR amplification, restriction enzyme (NEB and TaKaRa) digestion, and T4 DNA ligase ligation (Thermo Scientific, cat. no. EL0011). T4 PNK (NEB, #M0201 V) and CIP (NEB, #M0290 V) were used to modify the terminals of DNA fragments. The first constructed vector was pRTVnRFP with an N-terminal TagRFP tag. Based on this original vector, a series of transient expression vectors with N-/C-terminal tags of HA, cMyc, EGFP or mVenus or without any tags were generated. Binary vectors for rice stable expression were also generated by adding the T-DNA, selection markers, as well as the replicon and stabilization sequences from *Agrobacterium tumefaciens*. The detailed scheme of vector construction is shown in Additional file 1: Additional Methods. A final list, maps, and sequence structure around the MCS for all of the vectors are provided in Additional file 1: Table S1, Figure S2, and Figure S3, respectively.

For testing the feasibility of using those vectors, three representative proteins, OsRac1 (LOC_Os01g12900) (Akamatsu et al. 2013; Ono et al. 2001), WRKY45 (LOC_Os05g25770) (Shimono et al. 2007), and SPIN6 (LOC_Os07g46450) (Liu et al. 2015), as well as the EGFP or TagRFP empty vector and luciferase (LUC) were chosen for fluorescence detection, Co-IP, and BiFC assay in rice protoplasts. The *OsRac1* gene was cloned through *Bam*HI and *Hin*dIII, generating pRTVnYFP-OsRac1, pRTVnGFP-OsRac1, pRTVnMyc-OsRac1, and pRTVnVC-OsRac1 constructs. The *WRKY45* CDS fragment was cloned through *Bam*HI and *Kpn*I, generating the pRTVnRFP-WRKY45 construct. The *SPIN6* was cloned through *Bam*HI and *Not*I, generating the pRTVnRFP-SPIN6, pRTVnHA-SPIN6, and pRTVnVN-SPIN6 constructs. The C terminal of luciferase, *cLUC*_*(417–568)*_, was amplified from pCAMBIA1300-cLUC (Chen et al. 2008) and inserted into pRTVnMyc through *Eco*RV. The LUC fragment was generated by overlap PCR from pCAMBIA1300-nLUC (Chen et al. 2008) and pCAMBIA1300-cLUC, and was ligated through *Bam*HI and *Hin*dIII restriction sites, generating the pRTVcVC-LUC construct. The primers used for construction are listed in Additional file 1: Table S2.

### Rice Protoplast Isolation and Transfection

Rice protoplast isolation and transfection were performed as described previously (Chen et al. 2006; He et al. 2016). About 3 μg of plasmid DNA was used to transfect ~ 2 × 10^5^ protoplasts by the PEG-mediated method. The fluorescent proteins or epitope-tagged proteins were detected at 14–20 h after transfection.

### Fluorescence Detection

Four plasmids, including pRTVnRFP-SPIN6, pRTVnGFP-OsRac1, pRTVnYFP-OsRac1, and pRTVnRFP-WRKY45, as well as the empty vectors pRTVnGFP and pRTVnRFP were used for protein localization and co-localization analysis in rice protoplasts. In the BiFC assay, plasmids pRTVnVN-SPIN6 with pRTVnVC-OsRac1 or pRTVcVC-LUC were co-expressed in rice protoplasts. The fluorescent proteins were observed with a confocal microscope (Carl Zeiss LSM T-PMT 880). The steps of fluorescence observation were executed according to the previous protocol (He et al. 2016). For observation of fluorescence in transgenic plants, the young leaf sheath tissue of pRHVnGFP transgenic T_0_ plants was used.

### Proteins Extraction, Co-IP, and Western Blot Detection

Procedures for protein extraction and Co-IP assay in the rice protoplast system were previously described (He et al. 2016). The plasmid pRTVnHA-SPIN6 was co-transfected with pRTVnMyc-OsRac1 or with the control, pRTVnMyc-cLUC. The anti-HA antibody (Roche, Cat. No. 11867423001) was used to purify the HA-tagged protein in the Co-IP assay. For detection of GFP protein in plants, proteins extracted from young leaves were used western blot detection with the anti-GFP antibody (Roche, Cat. No. 11814460001).

### Agrobacterium-Mediated Rice Transformation

For construction of the CRISPR/Cas9 plasmid pRHCas9-IPA1, the specific sequence primer pairs with the complementary PAM site of *IPA1* (LOC_Os08g39890) (Jiao et al. 2010) target sequence 5’-ccaCCGACTCGAGCTGTGCTCTC-3′ were synthesized, cloned to pEntry A, and then sub-cloned to pRHCas9. The detailed construction procedure of pRHCas9-IPA1 targeting rice *IPA1* is described in Additional file 1: Additional Methods. The pRHVnGFP empty vector and pRHCas9-IPA1 plasmids were introduced into *Agrobacterium* strain EHA105. Rice calli were induced from the mature seeds of NPB. Transgenic rice plants were generated by the *Agrobacterium*-mediated method (Qu et al. 2006).

### Genomic DNA Extraction for Genotyping

Rice genomic DNA was extracted by the cetyltrimethylammonium bromide (CTAB) method. To detect mutagenesis in the *IPA1* gene, target fragments were amplified with specific primer pairs *IPA1*-CSPID-F/ *IPA1*-CSPID-R (Additional file 1: Table S2) and were then sequenced.

## Additional file

Additional file 1: **Additional Methods.** **Figure S1.** Restriction maps for ubiquitin-1 and 35S promoters. **Figure S2.** Maps of all the 42 vectors. **Figure S3.** The sequence structures in the MCS region of the vectors. **Table S1.** List of the 42 vectors generated in this study. **Table S2.** List of all primers used in this paper. (PDF 1648 kb)
