# Correction to: A Versatile Vector Toolkit for Functional Analysis of Rice Genes

**DOI:** 10.1186/s12284-018-0238-x

**Published:** 2018-09-07

**Authors:** Feng He, Fan Zhang, Wenxian Sun, Yuese Ning, Guo-Liang Wang

**Affiliations:** 1 State Key Laboratory for Biology of Plant Diseases and Insect Pests, Institute of Plant Protection, Chinese Academy of Agricultural Sciences, Beijing, 100193 China; 2 College of Plant Protection, China Agricultural University, Beijing, 100193 China; 3 Department of Plant Pathology, The Ohio State University, Columbus, OH 43210 USA

## Correction

The caption of Fig. 5 contained an error. The updated caption along with the original figure is published in this correction article.

**Fig. 5:**
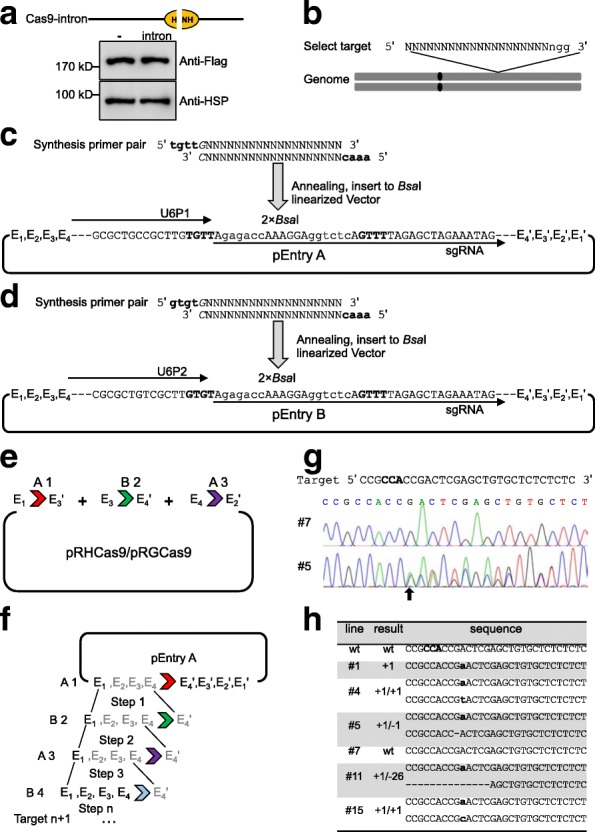
Diagram of how CRISPR/Cas9 vectors were constructed and used to edit the rice *IPA1* gene. **a** Western blot analysis of the *Cas9-intron* expression in rice protoplasts. HSP indicates the loading amount of each sample. **b** Target site selection for candidate genes in the rice genome. A 20-bp specific sequence followed by the PAM “NGG” structure is required. **c** and **d** Target cloning to the entry vectors. Synthesis of the primer pairs of the 20-bp specific target with the 4-bp adapters, and ligation with the *Bsa*I linearized pEntry A or pEntry B vector. **e** One-step ligation and **f** step-by-step ligation of multiple targets to pRHCas9/pRGCas9. Four pairs of isocaudamers, *Pst*I(E1)-*Nsi*I(E1'), *Xba*I(E2)-*Spe*I(E2'), *Bam*HI(E3)-*Bgl*II(E3'), and *Sal*I(E4)-*Xho*I(E4') are marked. The sgRNA cassettes with U6P1 and U6P2 in pEntry A and B should be used in turn. **g** Representative sequencing chromatogram of the CRISPR-*IPA1* transgenic lines. Line #7, wild-type genotype; line #5, mutant genotype. **h** Representative gene editing results in the CRISPR-*IPA1* transgenic lines

Table S1 in the Additional file of this original publication contained some errors. The updated Table S1 is published in this correction article.

## Additional file

Additional file 1: Updated Table S1, the full supplementary materials can be downloaded from the original publication. (XLSX 10 kb)
