# Supplementary material for: A Versatile Vector Toolkit for Functional Analysis of Rice Genes
Source: Rice (N Y). 2018 Apr 20;11:27. doi: 10.1186/s12284-018-0220-7 (PMC5910328; doi:10.1186/s12284-018-0220-7)
Supplement: Supplementary file 1 — Additional Methods. Figure S1. Restriction maps for ubiquitin-1 and 35S promoters. Figure S2. Maps of all the 42 vectors. Figure S3. The sequence structures in the MCS region of the vectors. Table S1. List of the 42 vectors generated in this study. Table S2. List of all primers used in this paper. (PDF 1648 kb) [file 12284_2018_220_MOESM1_ESM.pdf]

Additional file 1

Additional Methods

Part 1 Plasmid vectors construction

Construction of the first vector pRTVnRFP. To start the construction, four DNA fragments were used to form a circle plasmid in one step ligation. These four fragments included the adjacent high copy number *E.coli* pUC replicon and kanamycin screening gene from pDBLeu vector (Yeast-Two-Hybrid system, CAT. SERIES 10835, Invitrogen), the *Nos* terminator (NosT) from pCXUN (GenBank: FJ905215.1), the tagRFP (GenBank: EF606900.1) from pYBA1157 (GenBank: KF876809.1) and the strong maize ubiquitin-1 (Ubi) promoter with intron from pCXUN, which were amplified with primer pairs P1/ P2-3, P3/ P4-3, P11/ P12 and P18/ P19-2, respectively. These fragments were digested and assembled through *Pst*I/ *Asc*I, *Asc*I/ *Bam*HI, *Bam*HI/ *Eco*RV, and *Eco*RV/ *Pst*I recognition sites. The final pRTVnRFP was obtained by substitute the NosT with 3 stop-codon-NosT PCR fragment with P3+/ P4-3 through *Not*I and *Asc*I recognition sites.

Construction of pRTVs overexpression vectors. To generate pRTVnHA, pRTVnMyc, pRTVnGFP and pRTVnYFP, the TagRFP tag in pRTVnRFP was substituted with HA, cMyc, GFP and mVenus, respectively. The coding DNA fragments of these tags were amplified (4×HA by P5/ P6; 4×cMyc by P7/ P8; GFP and mVenus by P9/ P10) and inserted to *Eco*RV/ *Bam*HI digested pRTVnRFP vector.

A primers pair (Adaptor F/ Adaptor R) was directly used to delete TagRFP sequence in pRTVnRFP, generating tag free vector pRTV. This was achieved by primers annealing and inserting to pRTVnRFP through *Eco*RV/ *Bam*HI sites. PCR products (4×HA by P5-2/ P6-2; 3×cMyc by P7-2/ P8-2; EGFP and mVenus by P9-2/ P10-2; tagRFP by P11-2/ P12-2) were digested with *Not*I and the isocaudamer *Eag*I, then inserted to *Not*I and Calf Intestinal Alkaline Phosphatase (CIP) (NEB, #M0290V) treated vector pRTV, generating C-terminal tag vectors pRTVcHA, pRTVcMyc, pRTVcGFP, pRTVcYFP, and pRTVcRFP. The correct direction of recombinants were selected by PCR and sequencing.

Construction of BiFC vectors. Yellow fluorescent protein mVenus was split at 155 amino acid residues and generated N-terminal mVenus (mVN_{1-155}) and C-terminal mVenus ($mVC_{156-238}$) for BiFC assay. Before constructing the N or C-terminal mVenus to the vector, another independent fluorescent protein expression element (35S-ECFP-polyA or 35S-mCherry-polyA), was inserted to pRTV. These two elements were generated by overlapping PCR method. First, PCR amplify the 35S (P34-2/ P25) from pYBA1152, ECFP and mCherry (P32/ P33) from pYBA1154 and pYBA1158, and polyA (P26/ P27-3) from pCXUN. Then, 3 PCR fragments were used as template in secondary PCR with primers P34-2/ P27-3. The large PCR products 35S-ECFP-polyA or 35S-mCherry-polyA was purified and digested with *Nsi*I, followed by ligation with *Pst*I and CIP treated pRTV, generating the intermediate vectors pRTV-35S-ECFP and pRTV-35S-mCherry.

Finally, the mVN_{1-155}-HA or HA-mVN_{1-155} was amplified (mVN_{1-155}-HA by P9/ P13, HA-mVN_{1-155} by P9-3/ P13-2) from pYBA1155 vector and digested with *Eco*RV/ *Bam*HI or *Not*I/ *Eag*I, then inserted to pRTV-35S-ECFP through *Eco*RV/ *Bam*HI or *Not*I sites, generating N-terminal tag vector pRTVnVN and C-terminal tag vector pRTVcVN, respectively. Likewise, the $mVC_{156-238}$-cMyc or cMyc-$mVC_{156-238}$ was amplified ($mVC_{156-238}$-cMyc by P14-2/ P15, cMyc-$mVC_{156-238}$ by P14-3/ P10-2) and inserted to pRTV-35S-mCherry through *Eco*RV/ *Bam*HI or *Not*I sites, generating N-terminal tag vector pRTVnVC and C-terminal tag vector pRTVcVC, respectively.

Construction of pRHVs and pRGVs plant overexpression vectors. The pRHVs and pRGVs plant overexpression vectors were designed for transgenic overexpression with Hygromycin and G418/ Kanamycin selection marker, respectively. To generate pRHV and pRGV, two DNA fragments were PCR amplified and inserted into pRTV in step by step manner. Step 1 was to insert the *Not*I site mutated fragment for replication in *Agrobacterium* and the T-DNA right border (i.e. pVS1 STA-REP-ori-RB) to pRTV. Two pairs of primers P20-4/ P21MR and P22MF/ P23-2 were used to amplify RB-pVS1ori-REP and pVS1STA from pCAMBIA1300 (GenBank: AF234296.1), respectively. The RB was synthesized in the primer P20-4. The two fragments were integrated by overlapping PCR, creating a point mutant *Not*I site (from GCGGCCGC to GCGTCCGC) between REP and STA, followed by

insertion to pRTV through *Asc*I site and generating the intermediate vector pRTV-RBpVS1. Step 2 was to generate LB-35S-hptII-PolyA or LB-35S-nptII-PolyA through PCR and insert it to pRTV-RBpVS1. LB-35S (P24-2/ P25 for pRHV, P24-3/ P25 for pRGV, from pYBA1152; LB was synthesized in the primer P24-2 and P24-3), hptII (P28/ P29, from pCAMBIA1300), nptII (P47-2/ P48, from pCAMBIA2300, GenBank: AF234315.1) and PolyA (P26/ P27-2, from pCXUN) were amplified independently. The PCR fragments were fused by overlapping PCR method and generating LB-35S-hptII-PolyA or LB-35S-nptII-PolyA. Then, the two fusion fragments was digested with *Nsi*I/ *Pst*I and inserted into *Pst*I and CIP treated pRTV-RBpVS1, generating the pRHV and pRGV vector, respectively.

pRHVnHA, pRHVnMyc and pRHVnGFP vectors were derived from pRHV by inserting the 4×HA, 4×cMyc and EGFP fragments from pRTVnHA, pRTVnMyc and pRTVnGFP through *Eco*RV/ *Bam*HI sites, respectively. While pRHVcHA/ pRGVcHA, pRHVcMyc/ pRGVcMyc, pRHVcGFP/ pRGVcGFP and pRHVcRFP/ pRGVcRFP were constructed by amplifying the fragments 4×HA(P5-2/ P6-2), 3×/ 4×cMyc (P7-2/ P8-2), EGFP (P9-2/ P10-2) and TagRFP (P11-2/ P12-2) and inserting into pRHV and pRGV through *Not*I site, respectively.

Construction of pRTEs, pRHEs and pRGEs vectors without the Ubi promoter. A pair of oligos P49/ P50 was used to delete the strong Ubi promoter through *Pst*I/ *Eco*RV, turning the vectors pRTV, pRTVcYFP, pRHV, pRHVcHA, pRHVcMyc, pRGV, pRGVcHA and pRGVcMyc into pRTE, pRTEcYFP, pRHE, pRHEcHA, pRHEcMyc, pRGE, pRGEcHA and pRGEcMyc, respectively. Besides, the mVenus was amplified and inserted into pRHE and pRGE, leading to pRHEcYFP and pRGEcYFP, a similar procedure as the construction of pRTVcYFP.

Construction of CRISPR/Cas9 vectors. The rice codon optimized Cas9 sequence was amplified with P45/ P46 from pBY02-OsCas9-ccdB. To eliminate the Cas9 toxicity to *E.coli* and *Agrobacteria*, the second intron (IV2) of potato gene *ST-LS1* was amplified and inserted into Cas9 HNH nuclease domain. The Cas9 N-terminal (P45/ P53), intron (P51-2/ P52-2) and Cas9 C-terminal (P54/ P46) were amplified separately, and then fused by overlapping PCR with the primers P45/ P46. The Cas9-intron PCR fragment was digested and inserted into pRHV or pRGV through *Eco*RV/ *Not*I sites, generating intermediate vectors pRHV-Cas9 or

pRGV-Cas9, respectively. A pair of primer with MCS linker (P55/ P56) was designed and inserted into the intermediate vectors, generating the final CRISPR/ Cas9 binary vectors pRHCas9 and pRGCas9.

Meanwhile, the single guide RNA (sgRNA) was designed assembling in two entry vectors, named pEntry A and pEntry B. The sgRNA1 expression cassette (U6p1-sgRNA scaffold) was prepared by PCR fusion the U6p1 (P39-2/ P40-3) and sgRNA (P41-3/ P44-2) from pENTR4: gRNA4. While the sgRNA2 expression cassette (U6p2-sgRNA scaffold) was amplified directly from pENTR4: gRNA4 with P39-4/ P44-2. The two cassettes were inserted into pRTE through *Pst*I/ *Asc*I sites, generating pEntry A and pEntry B, respectively. Thus the entry vectors and binary vectors could be used for editing single or multiple targets s in rice.

Part 2 Construction of pRHCas9-IPA1 plasmid for genome editing.

Step 1. Synthesis the primer pair for *IPA1* target. The complementary PAM structure containing site of *IPA1* gene 5'-ccaCCGACTCGAGCTGTGCTCTC-3' was selected for sgRNA target. Synthesize primers IPA1-sgRNA-F: 5'-tgttGAGAGCACAGCTCGAGTCGG-3' and IPA1-sgRNA-R: 5'-aaacCCGACTCGAGCTGTGCTCTC-3';

Step 2. Preparation of double strand adaptor by primers annealing. Add the components and mix well as follow:

IPA1-sgRNA-F(10 μmol)	2 μl
IPA1-sgRNA-R(10 μmol)	2 μl
ddH_2O	16 μl
total	20 μl

Incubate in 70℃ for 10 min, place on ice immediately;

Step 3. Cloning Step 2 adaptor to pEntry A vector. Digest 1μg pEntry A vector by BsaI (NEB, #R0535V) in 37℃ for 2 h. Ligate the purified and digested pEntry A vector with the double strand adaptor in Step 2. Transform the ligation products into *E.coli* competent. Screen the recombinants with correct pEntry A-IPA1 by PCR and sequencing;

Step 4. Sub-cloning the IPA-sgRNA expression cassette to pRHCas9 vector. Digest pEntry A-IPA1 with *Pst*I/ *Spe*I, generating IPA-sgRNA expression cassette. Ligate the IPA-sgRNA expression cassette with PstI/ SpeI digested pRHCas9. After transformation, screen the recombinants by PCR, restriction enzymes digestion and sequencing.

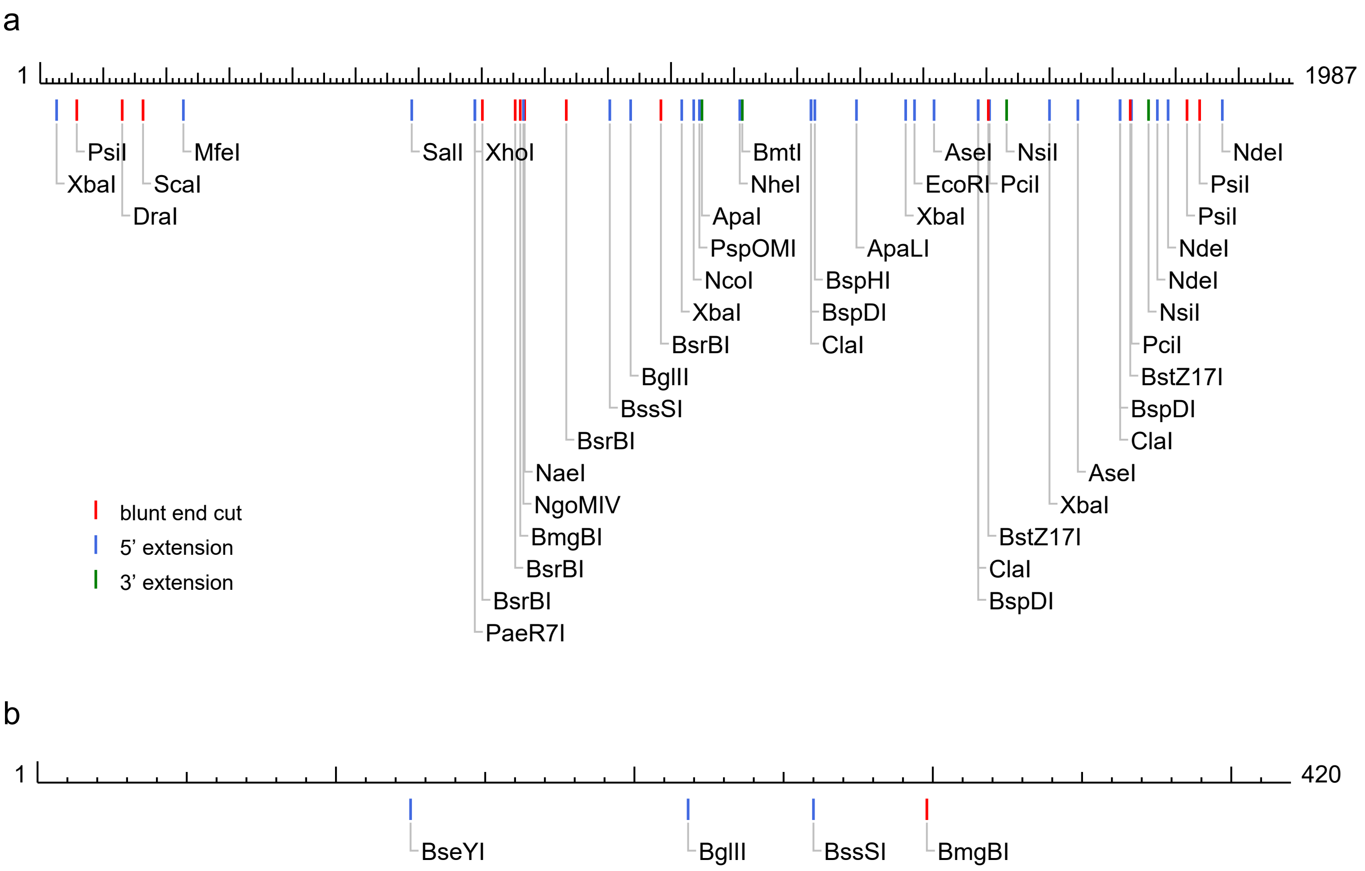

Figure S1. Restriction maps for ubiquitin-1 and 35S promoters

a Restriction enzyme sites in the ubiquitin-1 promoter. **b** Restriction enzyme sites in the 35S promoter. DNA sequences were analyzed with NEBcutter V2.0 (http://nc2.neb.com/NEBcutter2/). Only Type II and commercially available Type III enzymes with ⩾6 bp fixed recognition sequences are indicated.

Figure S2. Maps of all the 42 vectors

Vector size, sequence features and restriction sites of individual vector are included.

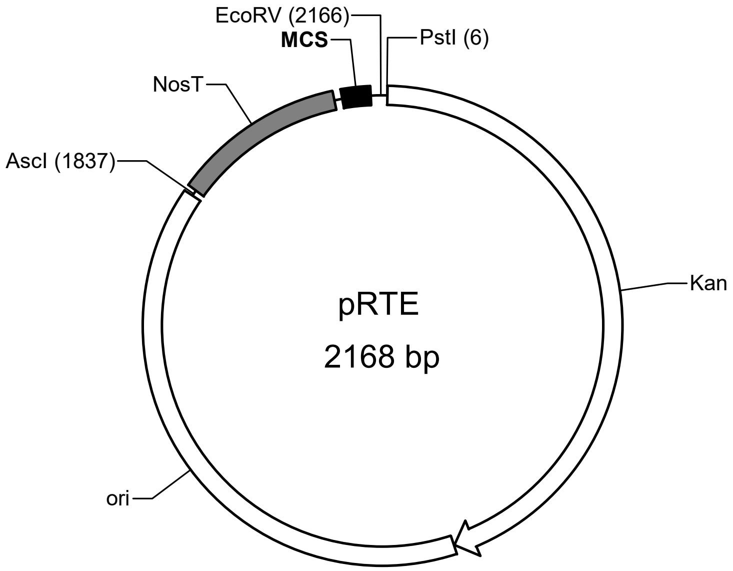
EcoRV (2166)
MCS
PstI (6)
NosT
AscI (1837)
Kan
pRTE
2168 bp
ori

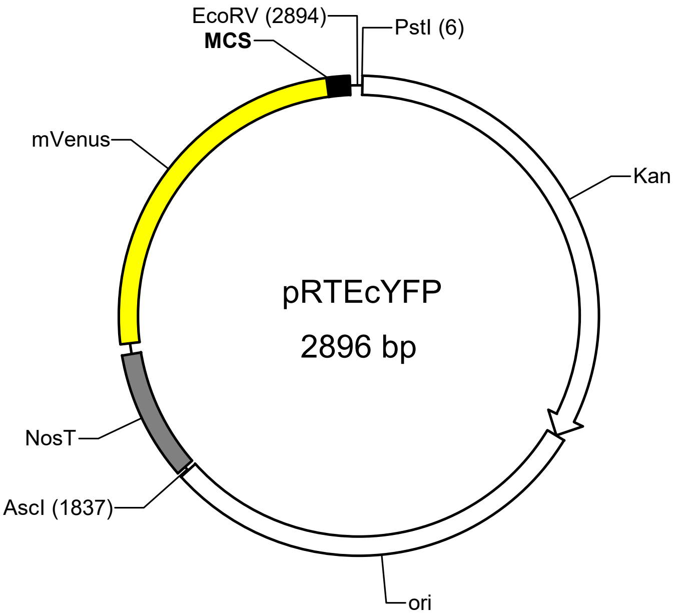
EcoRV (2894)
MCS
PstI (6)
mVenus
Kan
pRTEcYFP
2896 bp
NosT
AscI (1837)
ori

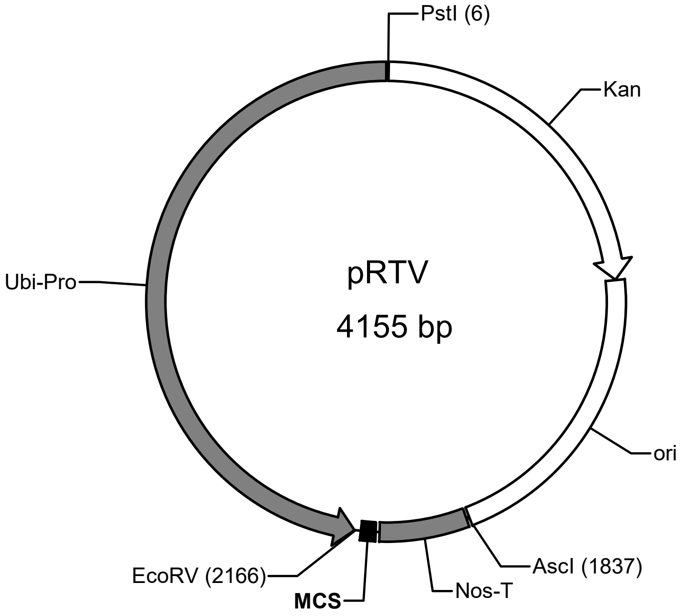
PstI (6)
Kan
pRTV
4155 bp
Ubi-Pro
ori
AscI (1837)
EcoRV (2166)
MCS
Nos-T

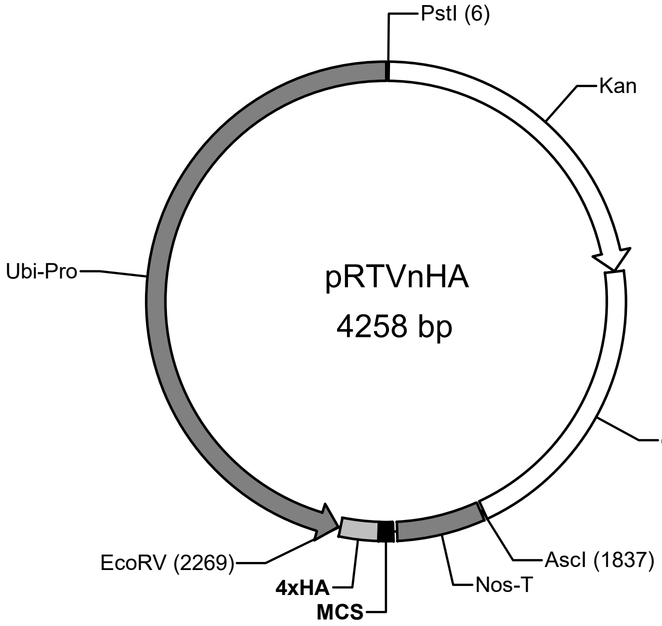
PstI (6)
Kan
ori
AscI (1837)
Nos-T
MCS
4xHA
EcoRV (2269)
Ubi-Pro
pRTVnHA
4258 bp

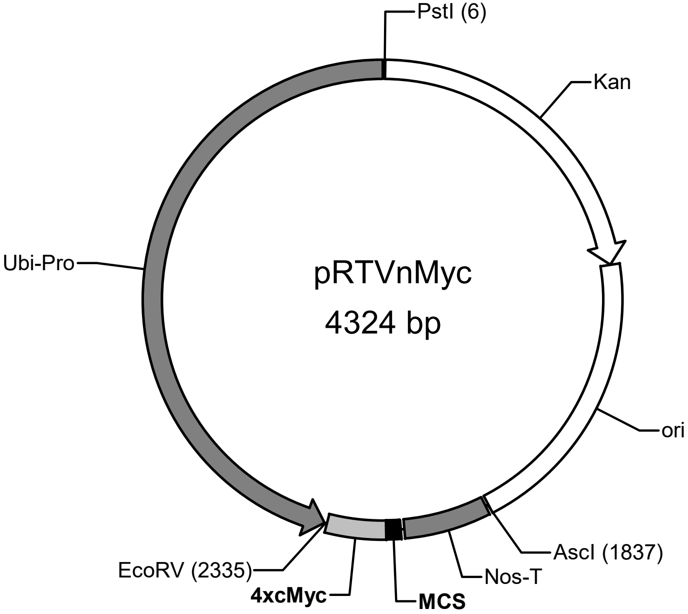
PstI (6)
Kan
Ubi-Pro
pRTVnMyc
4324 bp
ori
AscI (1837)
EcoRV (2335)
Nos-T
4xcMyc
MCS

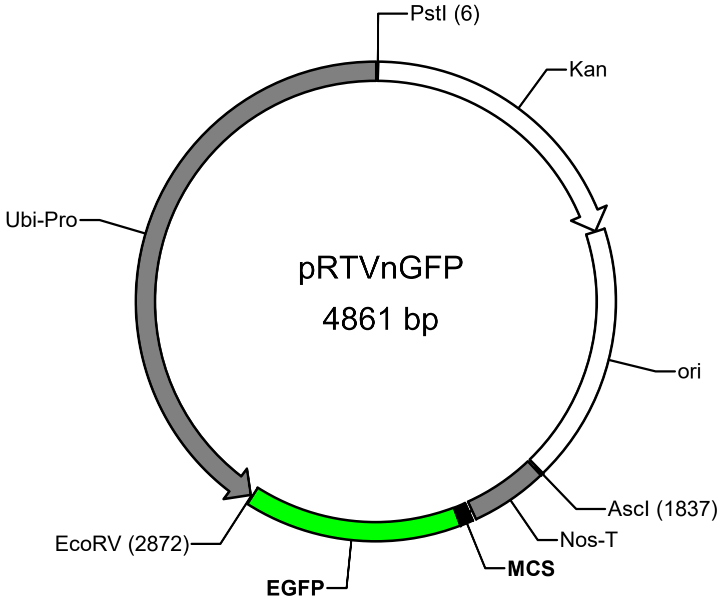
PstI (6)
Kan
Ubi-Pro
pRTVnGFP
4861 bp
ori
AscI (1837)
Nos-T
MCS
EGFP
EcoRV (2872)

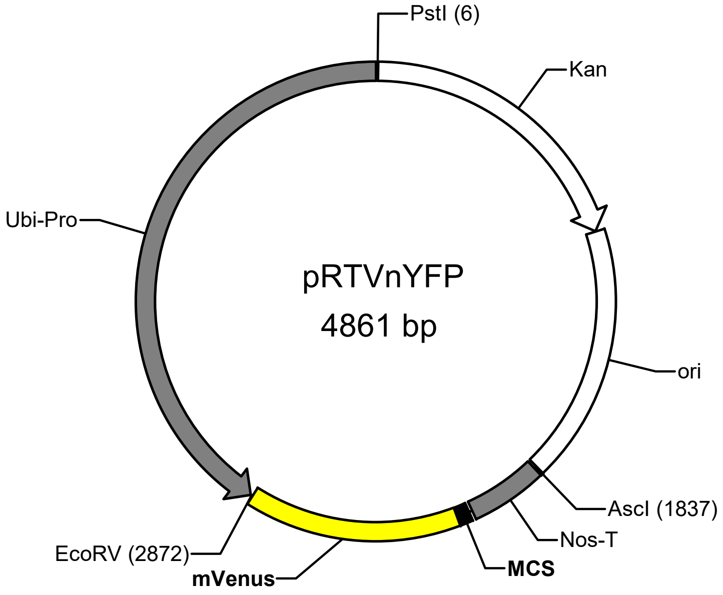
PstI (6)
Kan
Ubi-Pro
pRTVnYFP
4861 bp
ori
AscI (1837)
Nos-T
MCS
EcoRV (2872)
mVenus

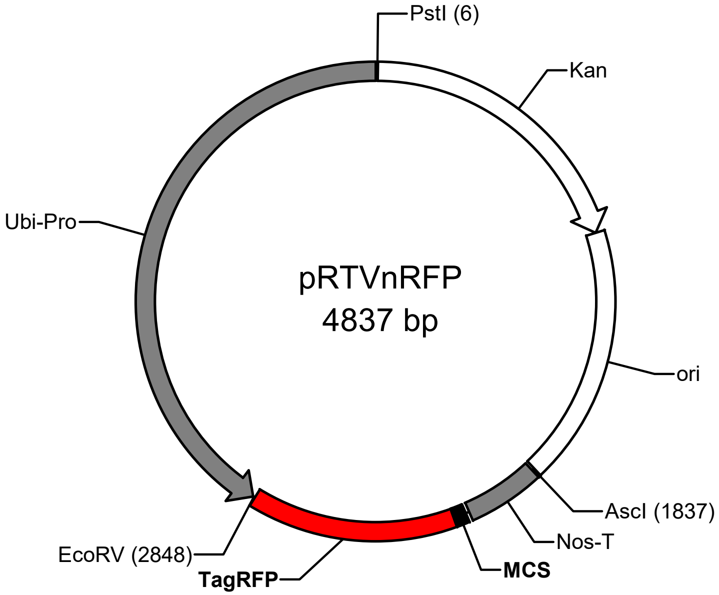
PstI (6)
Kan
Ubi-Pro
pRTVnRFP
4837 bp
ori
AscI (1837)
Nos-T
EcoRV (2848)
MCS
TagRFP

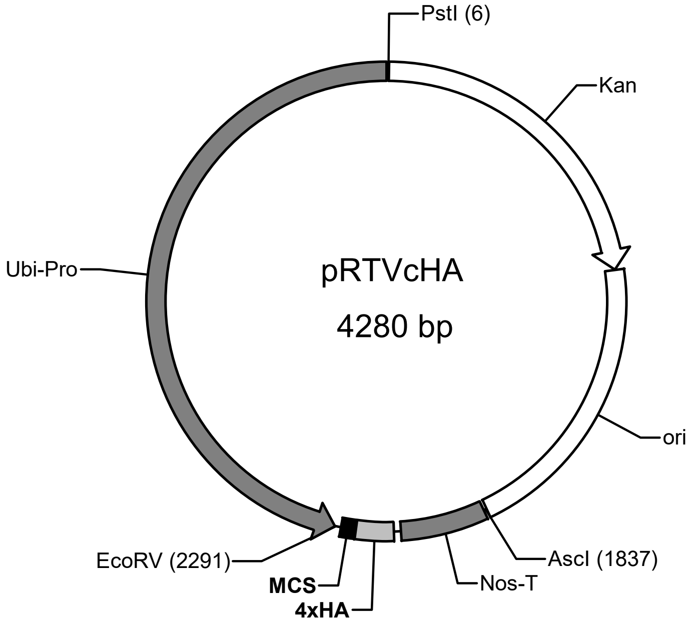
PstI (6)
Kan
pRTVcHA
4280 bp
Ubi-Pro
ori
EcoRV (2291)
AscI (1837)
Nos-T
MCS
4xHA

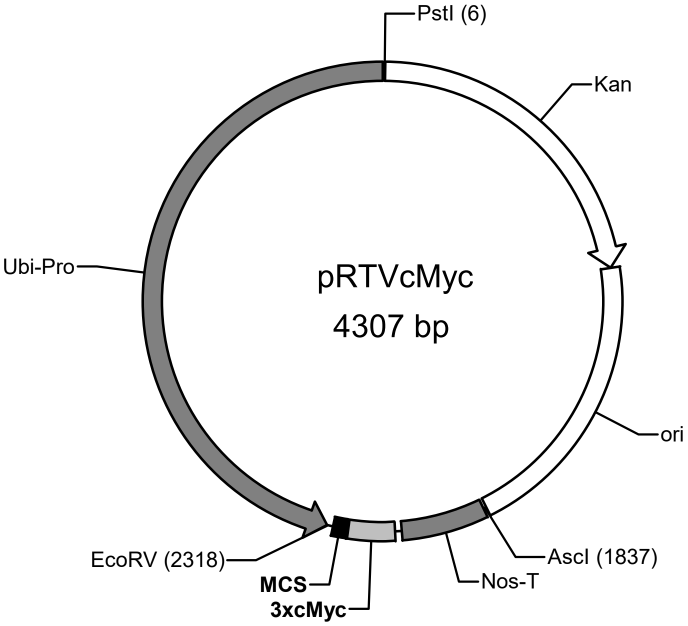
PstI (6)
Kan
Ubi-Pro
pRTVcMyc
4307 bp
ori
EcoRV (2318)
MCS
3xcMyc
Nos-T
AscI (1837)

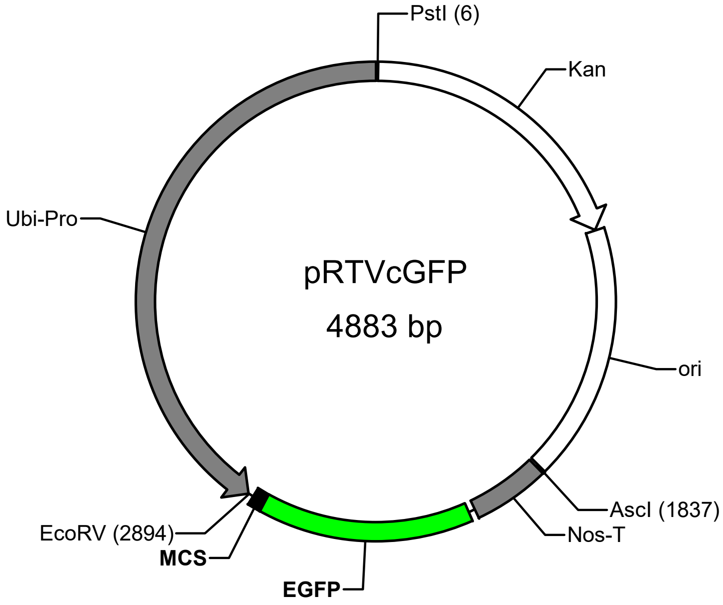
PstI (6)
Kan
Ubi-Pro
pRTVcGFP
4883 bp
ori
AscI (1837)
Nos-T
EcoRV (2894)
MCS
EGFP

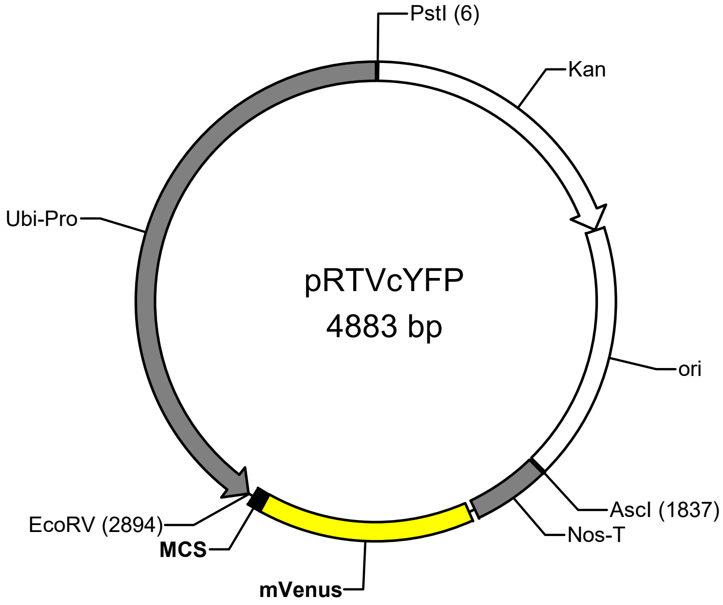
PstI (6)
Kan
Ubi-Pro
pRTVcYFP
4883 bp
ori
AscI (1837)
Nos-T
EcoRV (2894)
MCS
mVenus

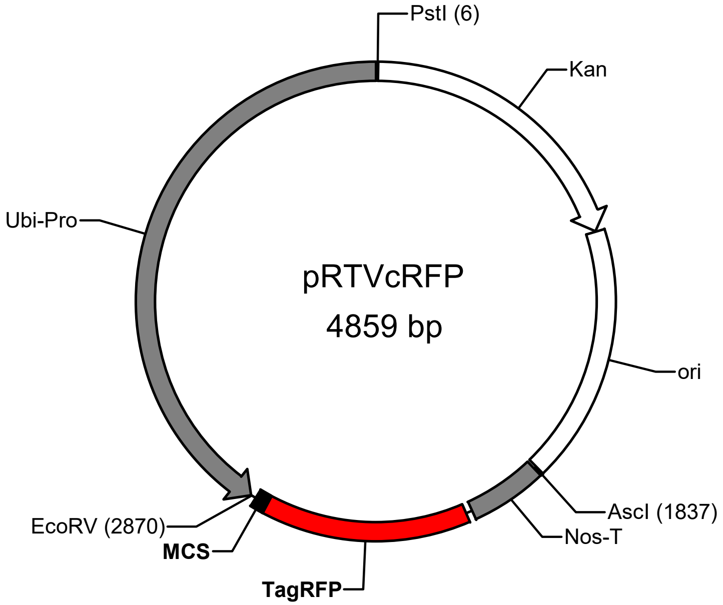
PstI (6)
Kan
Ubi-Pro
pRTVcRFP
4859 bp
ori
AscI (1837)
Nos-T
EcoRV (2870)
MCS
TagRFP

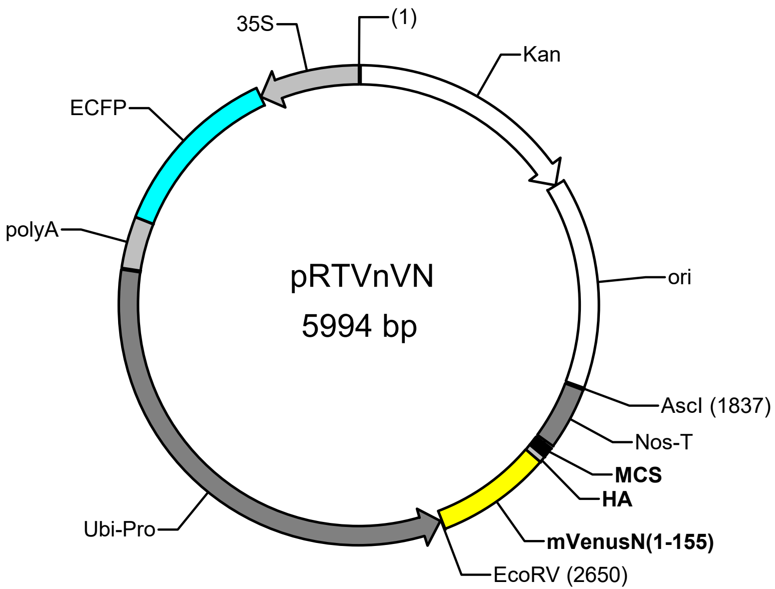

pRTVnVN
5994 bp
(1)
35S
Kan
ECFP
polyA
ori
AscI (1837)
Nos-T
MCS
HA
mVenusN(1-155)
Ubi-Pro
EcoRV (2650)

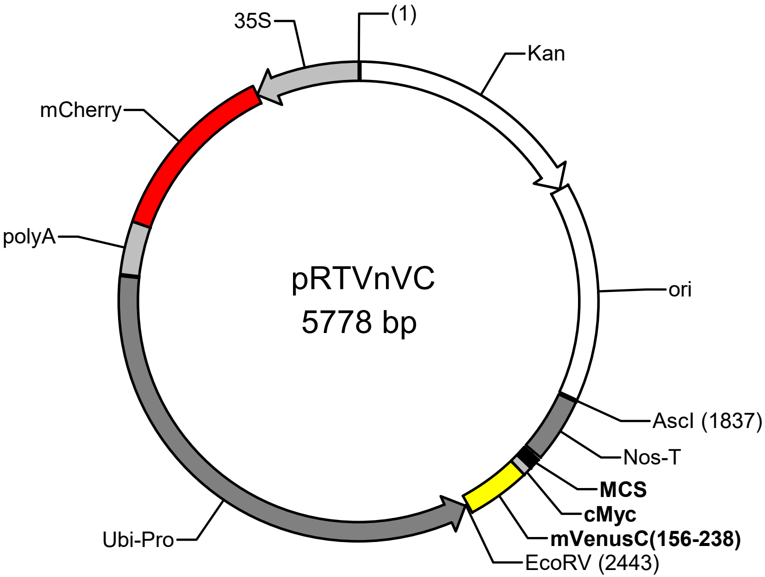

35S
(1)
Kan
mCherry
polyA
pRTVnVC
5778 bp
ori
AscI (1837)
Nos-T
MCS
cMyc
mVenusC(156-238)
EcoRV (2443)
Ubi-Pro

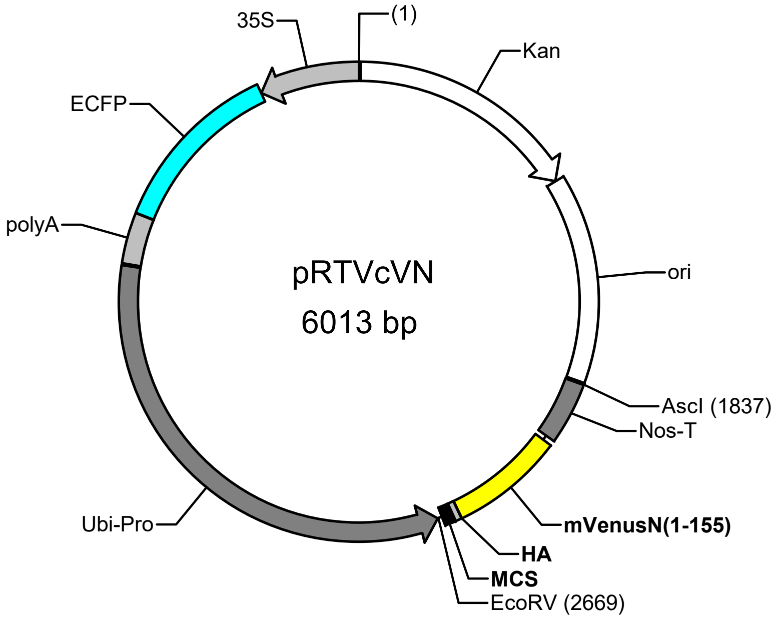

(1)
35S
Kan
ECFP
polyA
ori
pRTVcVN
6013 bp
AscI (1837)
Nos-T
mVenusN(1-155)
Ubi-Pro
HA
MCS
EcoRV (2669)

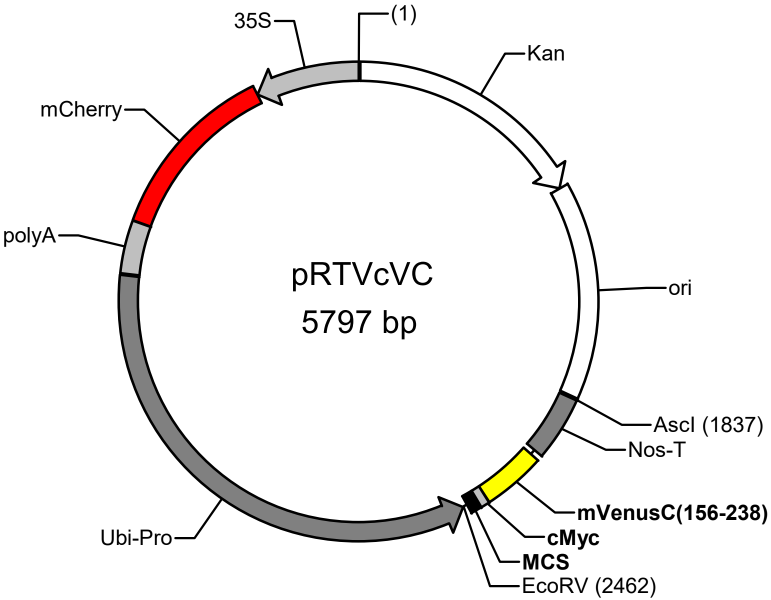
pRTVcVC
5797 bp
(1)
35S
Kan
mCherry
polyA
ori
AscI (1837)
Nos-T
mVenusC(156-238)
cMyc
MCS
EcoRV (2462)
Ubi-Pro

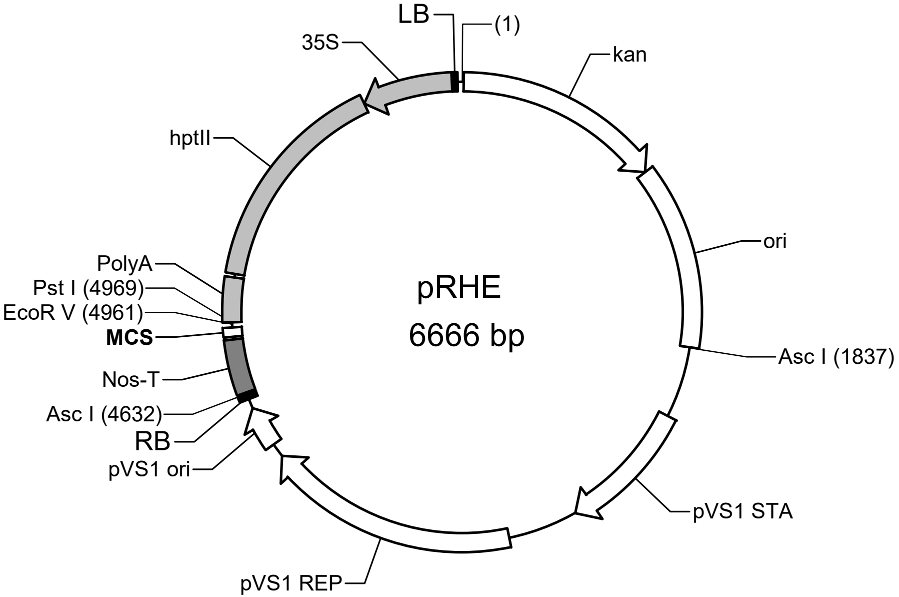

LB
(1)
35S
kan
hptII
ori
PolyA
Pst I (4969)
EcoR V (4961)
MCS
pRHE
6666 bp
Asc I (1837)
Nos-T
Asc I (4632)
RB
pVS1 ori
pVS1 STA
pVS1 REP

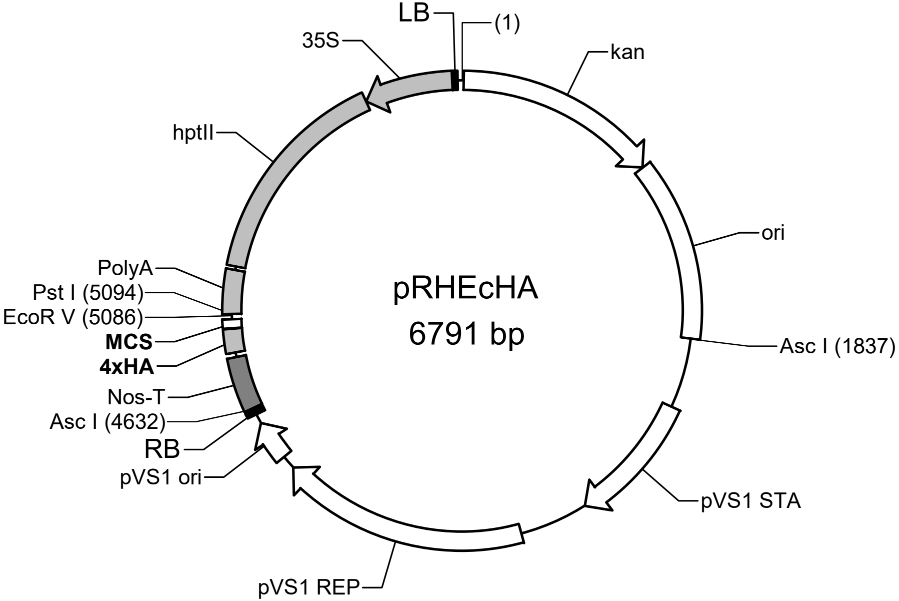
pRHEcHA
6791 bp
LB
(1)
kan
ori
Asc I (1837)
pVS1 STA
pVS1 REP
pVS1 ori
RB
Asc I (4632)
Nos-T
4xHA
MCS
EcoR V (5086)
Pst I (5094)
PolyA
hptII
35S

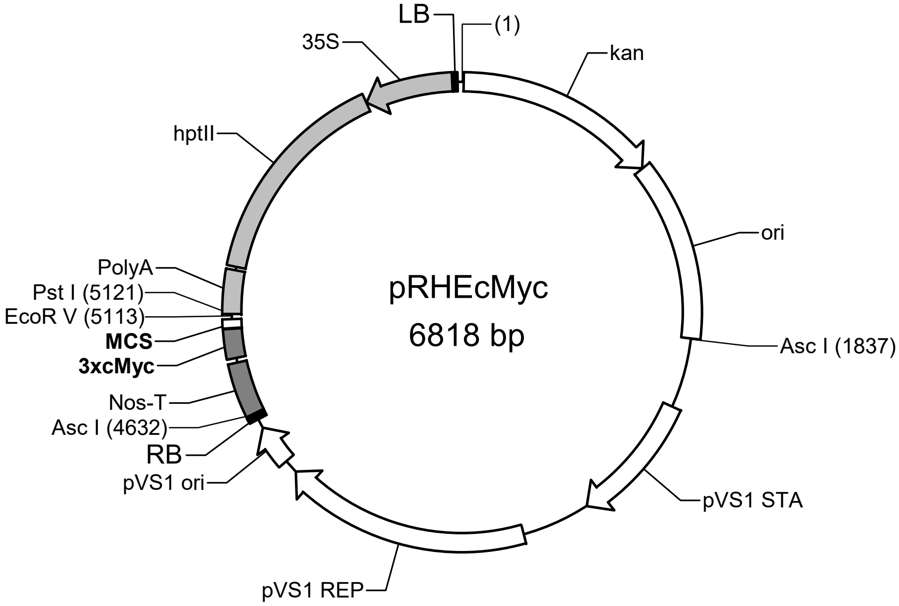
pRHEcMyc
6818 bp
LB
(1)
kan
ori
Asc I (1837)
pVS1 STA
pVS1 REP
pVS1 ori
RB
Asc I (4632)
Nos-T
3xcMyc
MCS
EcoR V (5113)
Pst I (5121)
PolyA
hptII
35S

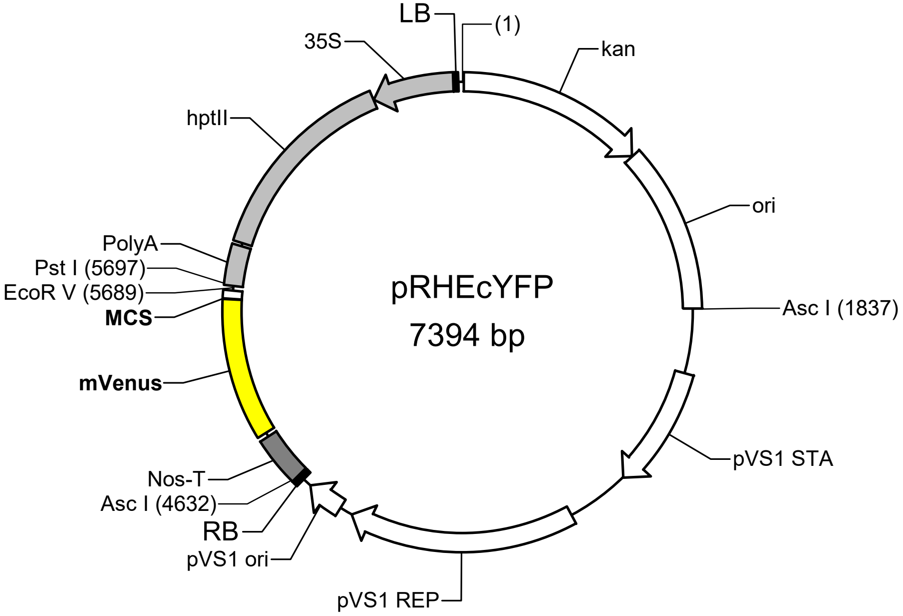

pRHEcYFP
7394 bp
LB
(1)
kan
35S
hptII
ori
Asc I (1837)
PolyA
Pst I (5697)
EcoR V (5689)
MCS
mVenus
pVS1 STA
Nos-T
Asc I (4632)
RB
pVS1 ori
pVS1 REP

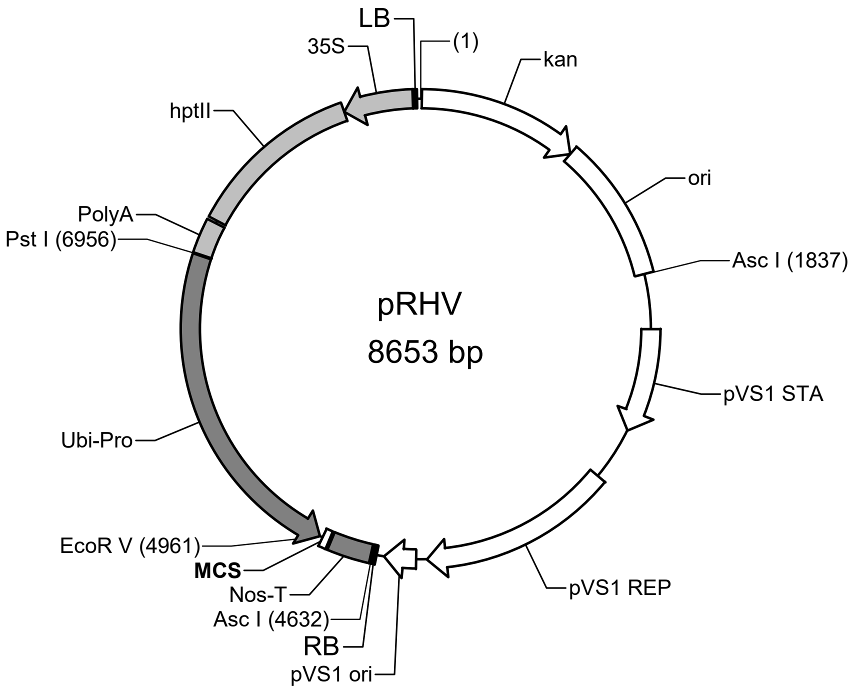

pRHV
8653 bp
LB
(1)
35S
kan
hptII
ori
Asc I (1837)
PolyA
Pst I (6956)
pVS1 STA
Ubi-Pro
EcoR V (4961)
MCS
Nos-T
Asc I (4632)
RB
pVS1 ori
pVS1 REP

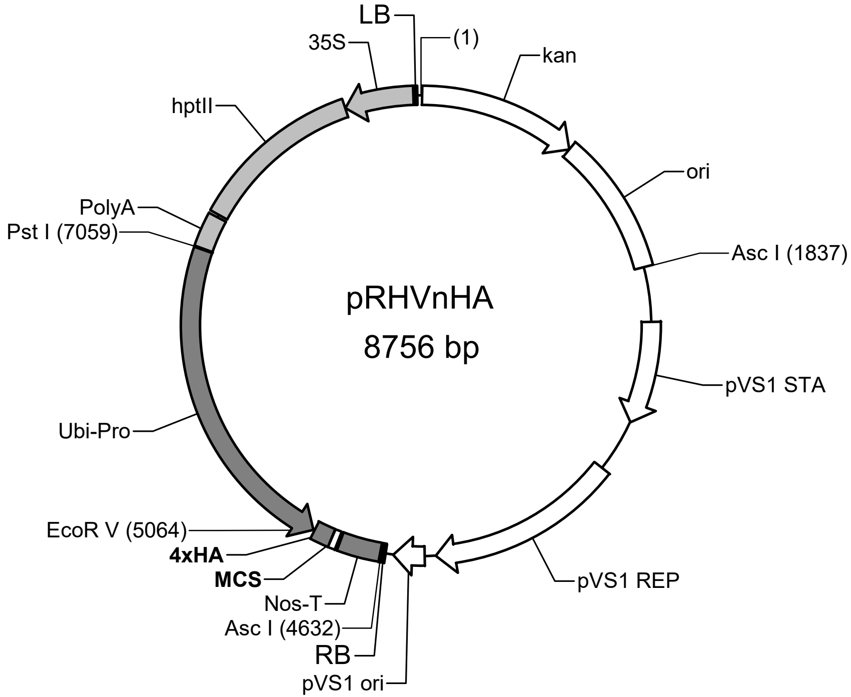
pRHVnHA
8756 bp
LB
(1)
35S
kan
hptII
ori
PolyA
Pst I (7059)
Asc I (1837)
pVS1 STA
Ubi-Pro
EcoR V (5064)
4xHA
MCS
Nos-T
Asc I (4632)
RB
pVS1 ori
pVS1 REP

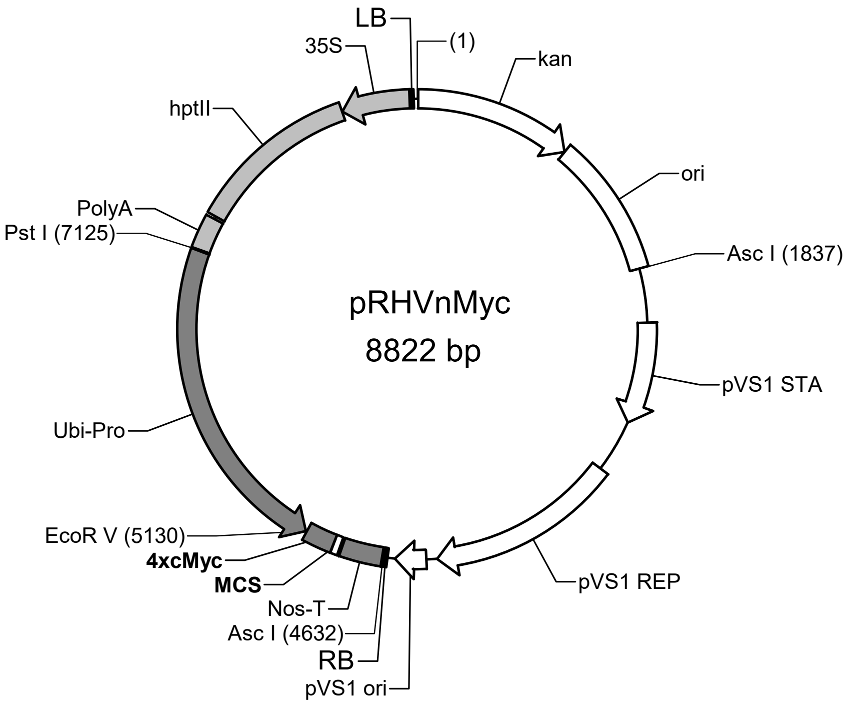
pRHVnMyc
8822 bp
LB
(1)
kan
35S
hptII
ori
Asc I (1837)
PolyA
Pst I (7125)
pVS1 STA
Ubi-Pro
pVS1 REP
EcoR V (5130)
4xcMyc
MCS
Nos-T
Asc I (4632)
RB
pVS1 ori

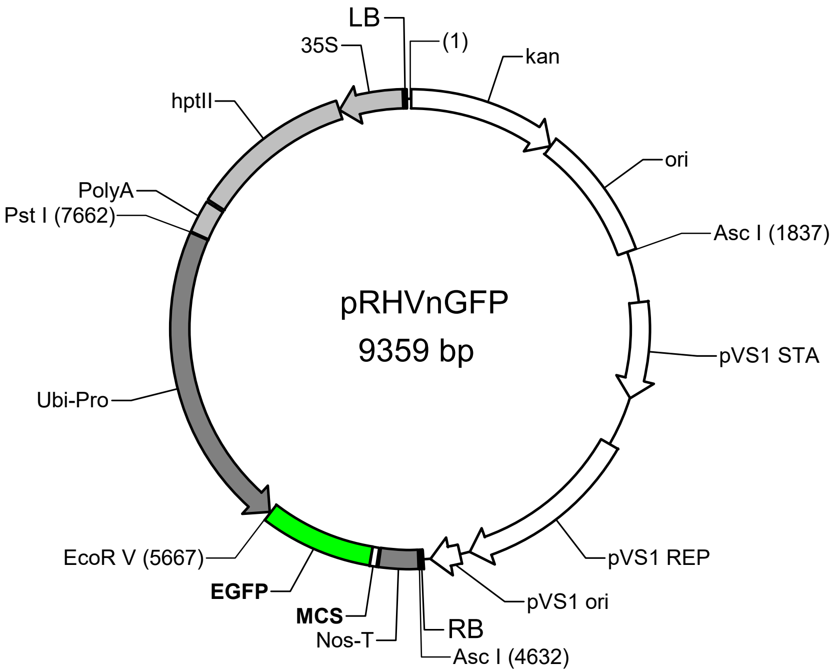

pRHVnGFP
9359 bp
LB
(1)
35S
kan
hptII
ori
PolyA
Pst I (7662)
Asc I (1837)
pVS1 STA
Ubi-Pro
pVS1 REP
EcoR V (5667)
EGFP
pVS1 ori
MCS
RB
Nos-T
Asc I (4632)

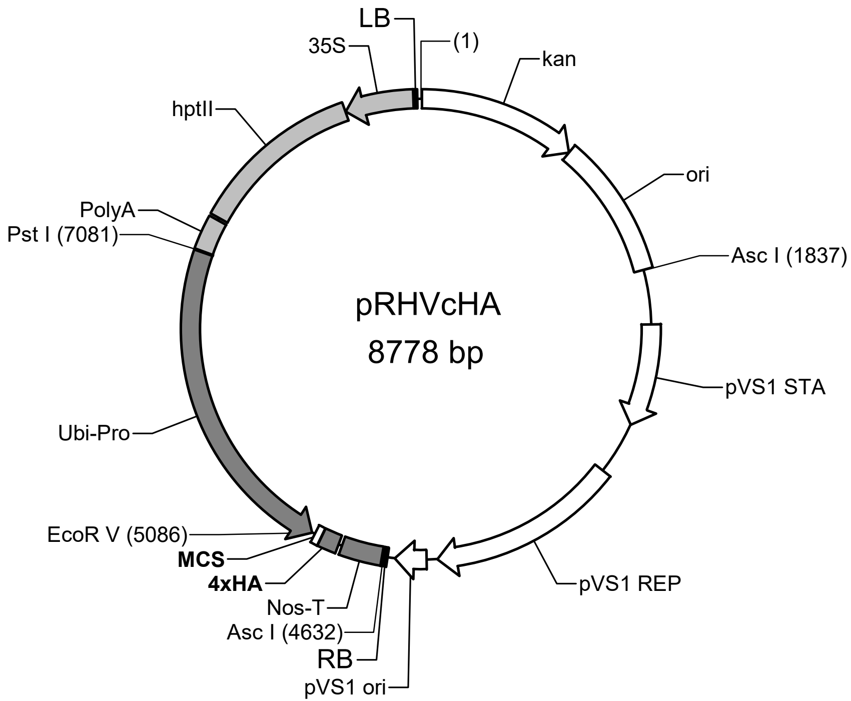

pRHVcHA
8778 bp
LB
(1)
35S
kan
hptII
ori
PolyA
Pst I (7081)
Asc I (1837)
pVS1 STA
Ubi-Pro
pVS1 REP
EcoR V (5086)
MCS
4xHA
Nos-T
Asc I (4632)
RB
pVS1 ori

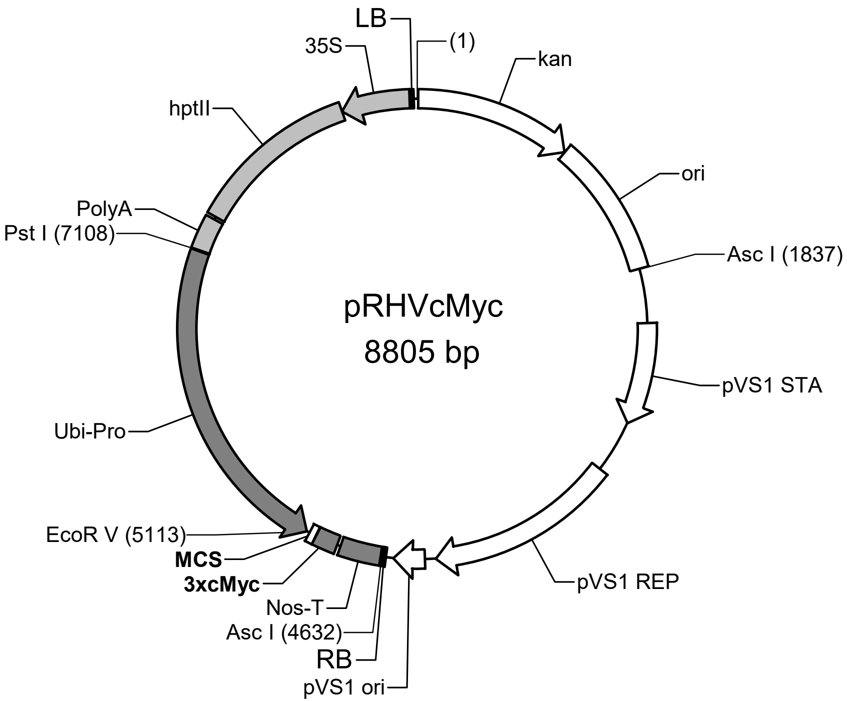
pRHVcMyc
8805 bp
LB
(1)
35S
kan
hptII
ori
PolyA
Pst I (7108)
Asc I (1837)
pVS1 STA
Ubi-Pro
EcoR V (5113)
MCS
3xcMyc
pVS1 REP
Nos-T
Asc I (4632)
RB
pVS1 ori

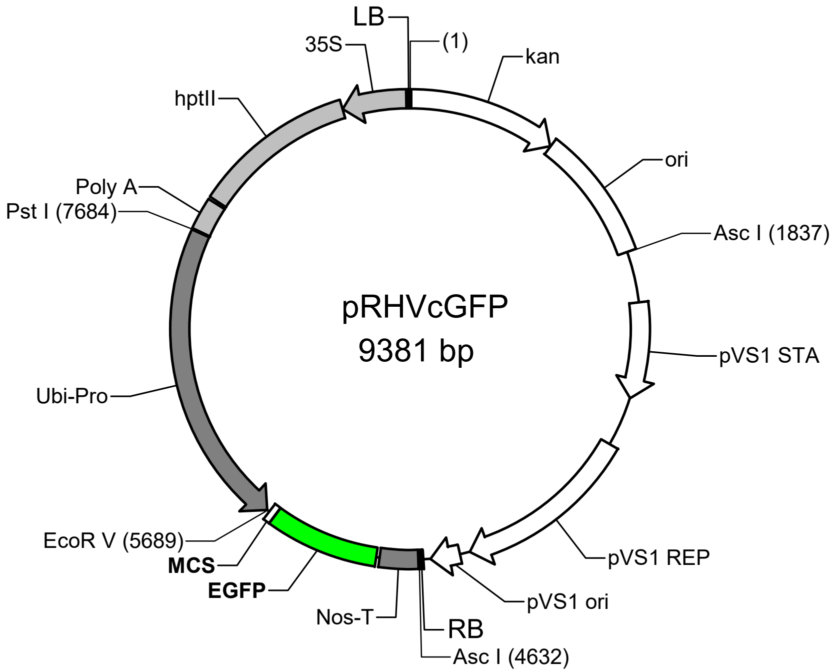

pRHVcGFP
9381 bp
LB
(1)
35S
kan
hptII
ori
Poly A
Pst I (7684)
Asc I (1837)
pVS1 STA
Ubi-Pro
EcoR V (5689)
pVS1 REP
MCS
EGFP
pVS1 ori
Nos-T
RB
Asc I (4632)

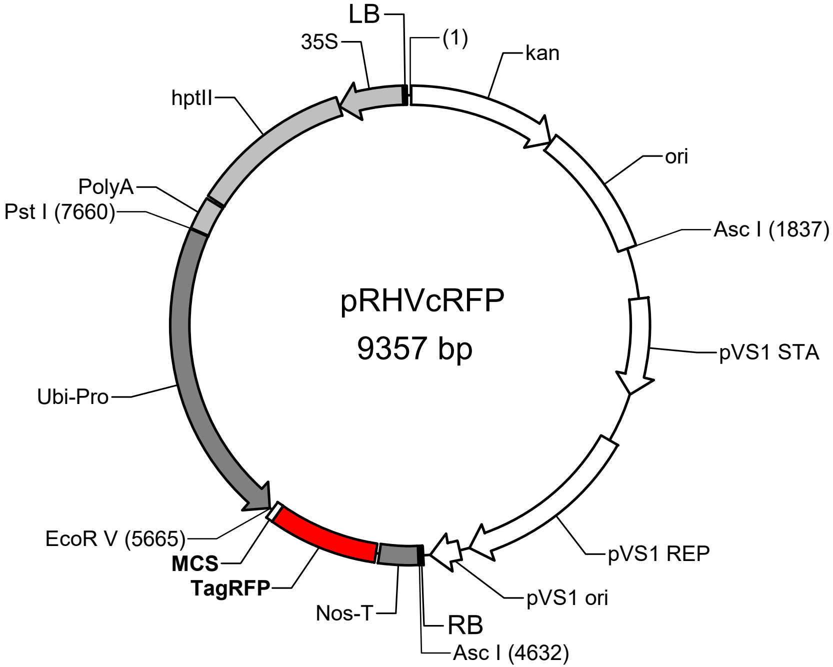
pRHVcRFP
9357 bp
LB
(1)
35S
kan
hptII
ori
PolyA
Pst I (7660)
Asc I (1837)
pVS1 STA
Ubi-Pro
EcoR V (5665)
MCS
TagRFP
pVS1 REP
pVS1 ori
Nos-T
RB
Asc I (4632)

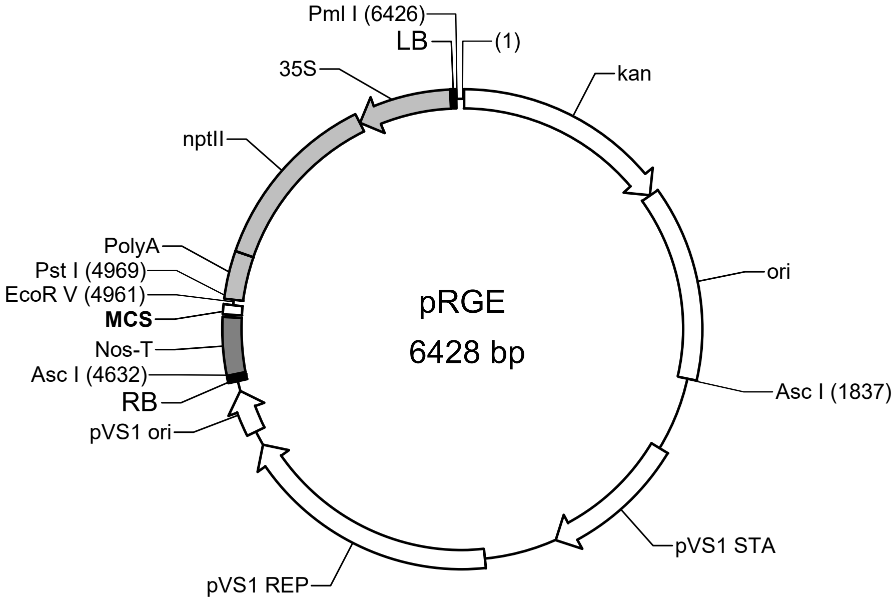

Pml I (6426)
LB
(1)
35S
kan
nptII
ori
PolyA
Pst I (4969)
EcoR V (4961)
MCS
Nos-T
Asc I (4632)
RB
pVS1 ori
Asc I (1837)
pVS1 STA
pVS1 REP
pRGE
6428 bp

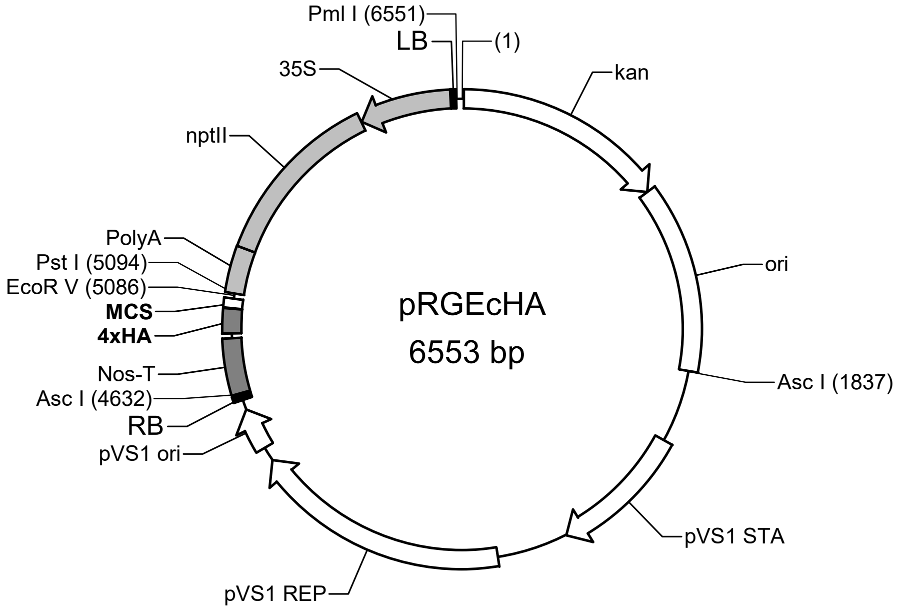

Pml I (6551)
LB
(1)
35S
kan
nptII
PolyA
Pst I (5094)
EcoR V (5086)
MCS
4xHA
Nos-T
Asc I (4632)
RB
pVS1 ori
ori
Asc I (1837)
pRGEcHA
6553 bp
pVS1 STA
pVS1 REP

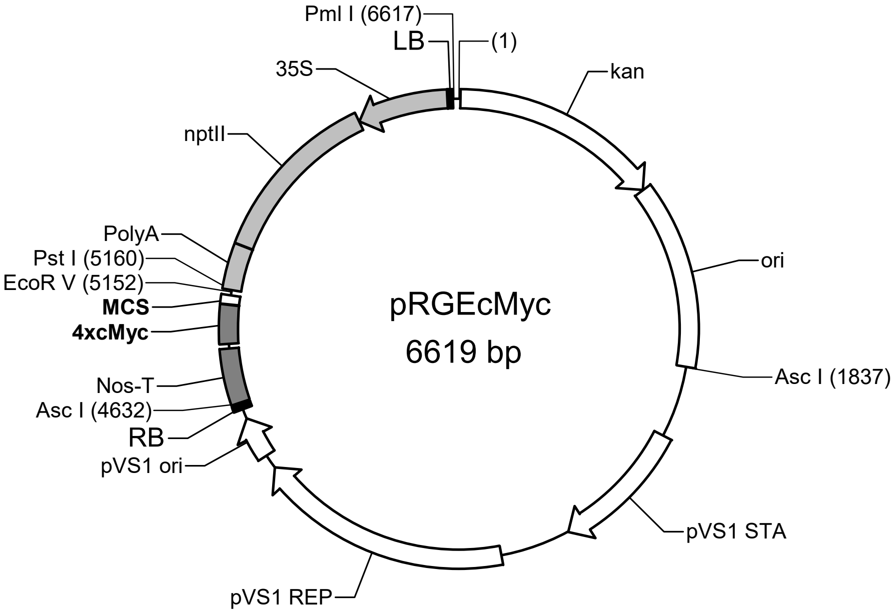
pRGEcMyc
6619 bp
Pml I (6617)
LB
(1)
kan
35S
nptII
ori
PolyA
Pst I (5160)
EcoR V (5152)
MCS
4xcMyc
Asc I (1837)
Nos-T
Asc I (4632)
RB
pVS1 ori
pVS1 STA
pVS1 REP

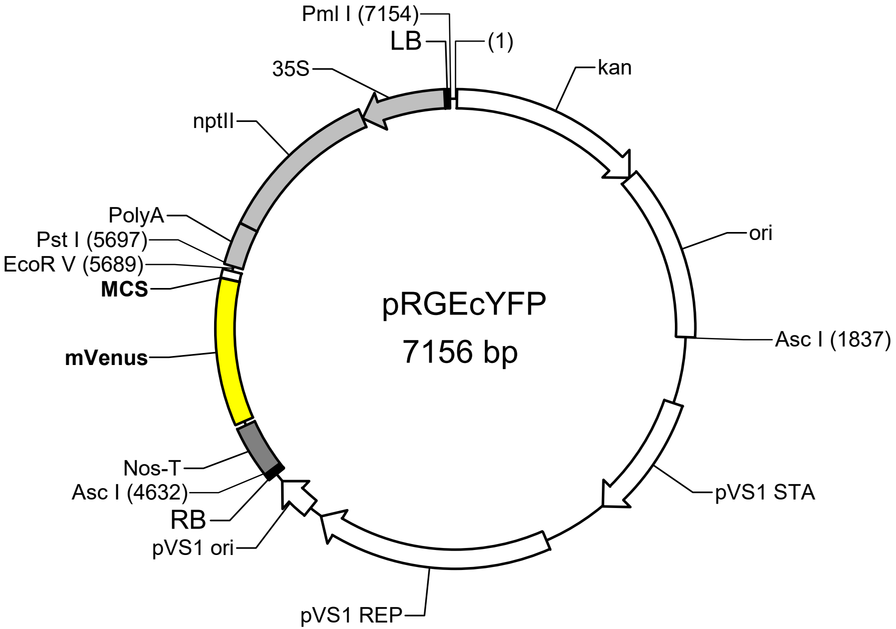

Pml I (7154)
LB
(1)
35S
kan
nptII
PolyA
Pst I (5697)
EcoR V (5689)
MCS
ori
pRGEcYFP
7156 bp
Asc I (1837)
mVenus
Nos-T
Asc I (4632)
RB
pVS1 ori
pVS1 STA
pVS1 REP

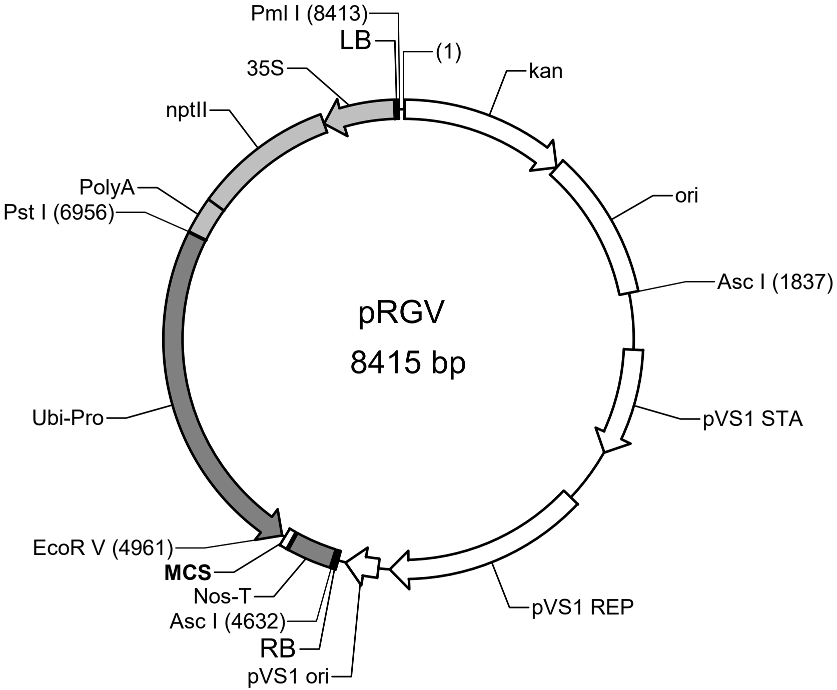

pRGV
8415 bp
Pml I (8413)
LB
(1)
35S
kan
nptII
ori
PolyA
Pst I (6956)
Asc I (1837)
Ubi-Pro
pVS1 STA
EcoR V (4961)
MCS
Nos-T
Asc I (4632)
RB
pVS1 ori
pVS1 REP

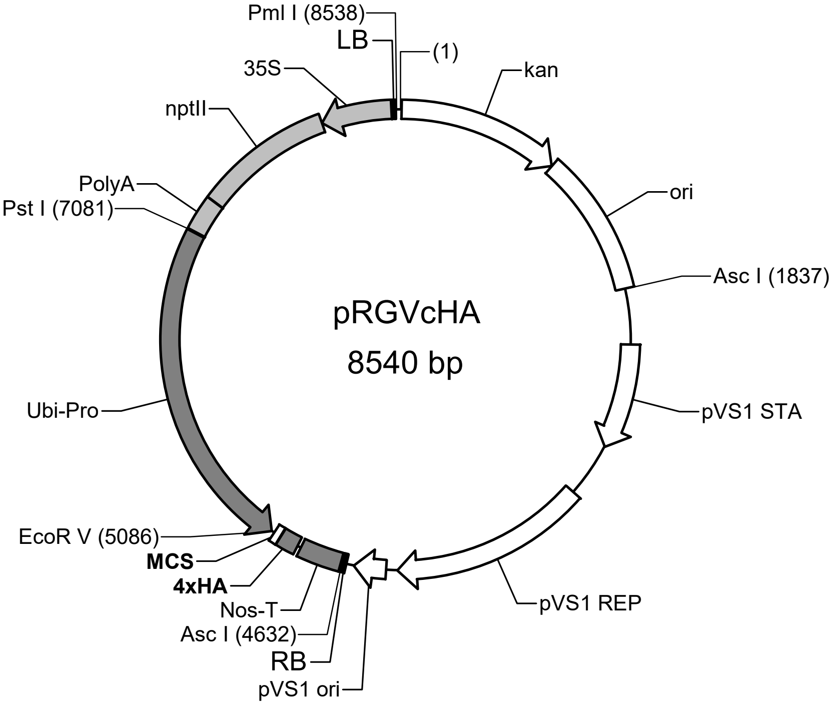
pRGVcHA
8540 bp
Pml I (8538)
LB
(1)
kan
35S
nptII
ori
PolyA
Pst I (7081)
Asc I (1837)
Ubi-Pro
pVS1 STA
EcoR V (5086)
MCS
4xHA
Nos-T
Asc I (4632)
RB
pVS1 ori
pVS1 REP

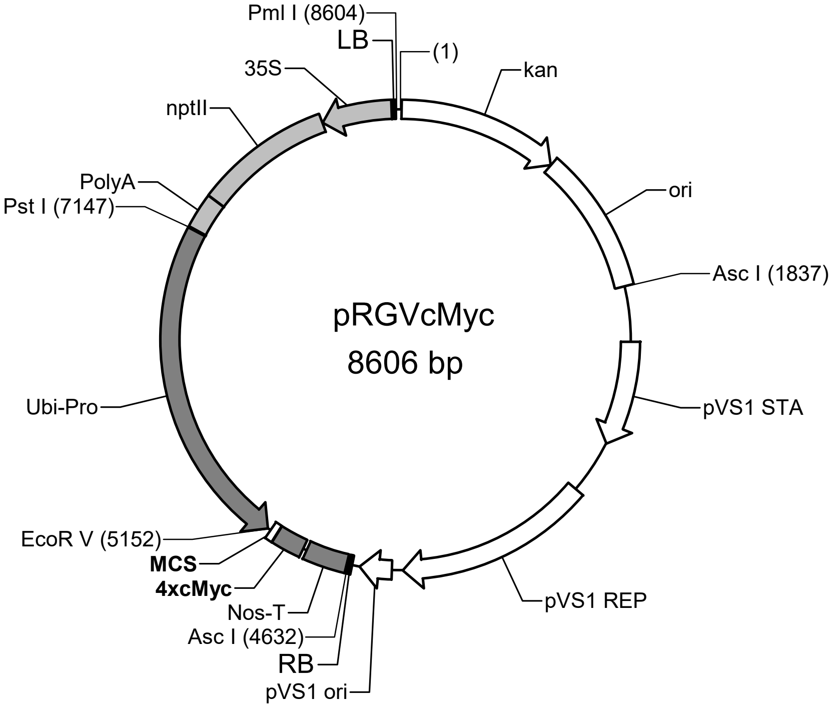

pRGVcMyc
8606 bp
Pml I (8604)
LB
(1)
35S
kan
nptII
ori
PolyA
Pst I (7147)
Asc I (1837)
Ubi-Pro
pVS1 STA
EcoR V (5152)
MCS
4xcMyc
Nos-T
Asc I (4632)
RB
pVS1 ori
pVS1 REP

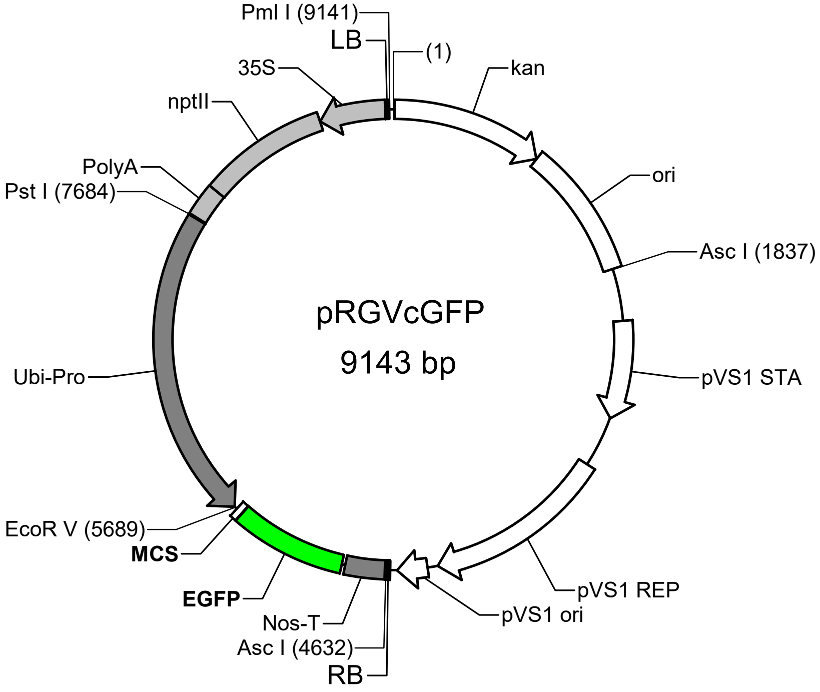
Pml I (9141)
LB
(1)
35S
kan
nptII
ori
PolyA
Pst I (7684)
Asc I (1837)
pRGVcGFP
9143 bp
Ubi-Pro
pVS1 STA
EcoR V (5689)
MCS
EGFP
Nos-T
Asc I (4632)
RB
pVS1 REP
pVS1 ori

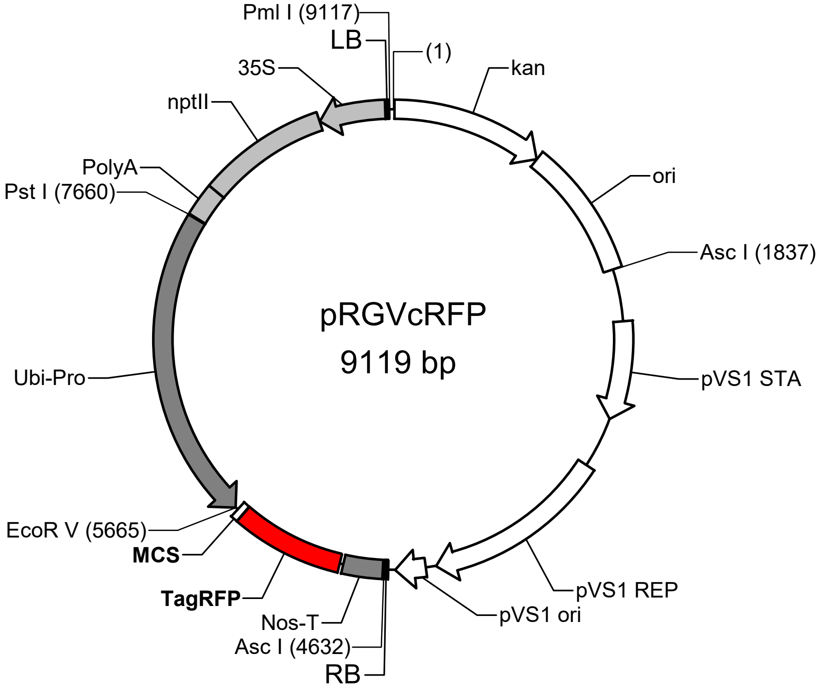
pRGVcRFP
9119 bp
Pml I (9117)
LB
(1)
kan
35S
nptII
PolyA
Pst I (7660)
ori
Asc I (1837)
Ubi-Pro
pVS1 STA
EcoR V (5665)
MCS
TagRFP
Nos-T
Asc I (4632)
RB
pVS1 REP
pVS1 ori

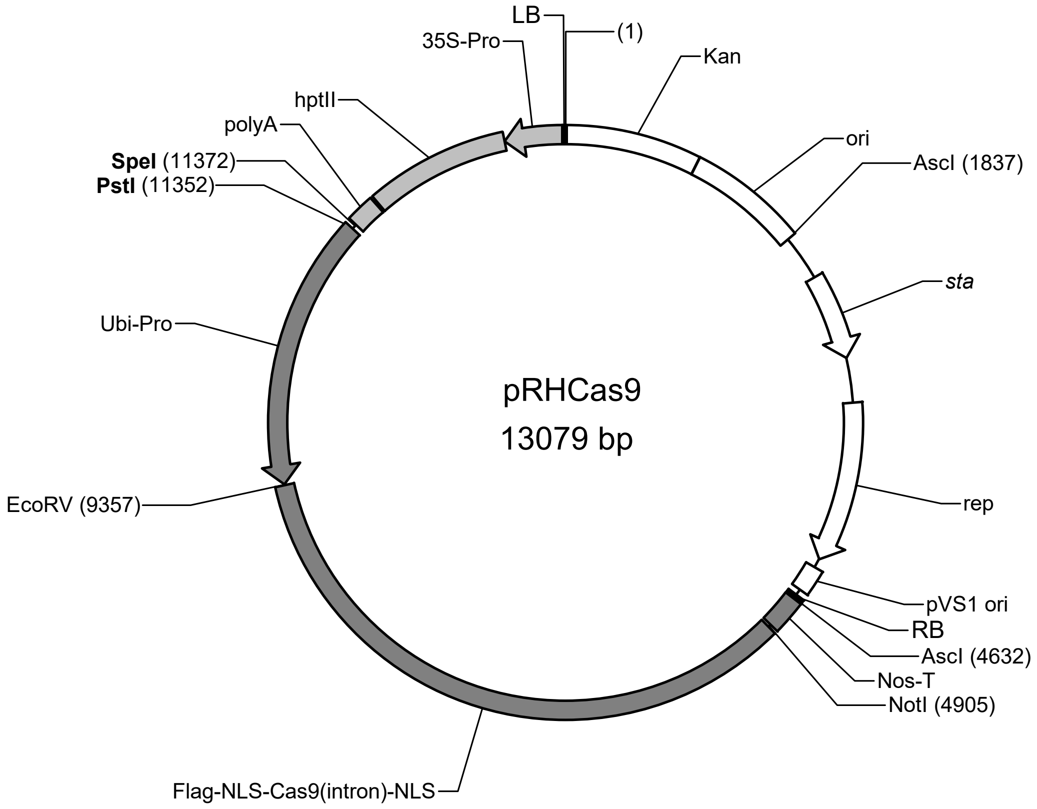

pRHCas9
13079 bp
LB
(1)
35S-Pro
Kan
hptII
ori
polyA
AscI (1837)
SpeI (11372)
PstI (11352)
sta
Ubi-Pro
rep
EcoRV (9357)
pVS1 ori
RB
AscI (4632)
Nos-T
NotI (4905)
Flag-NLS-Cas9(intron)-NLS

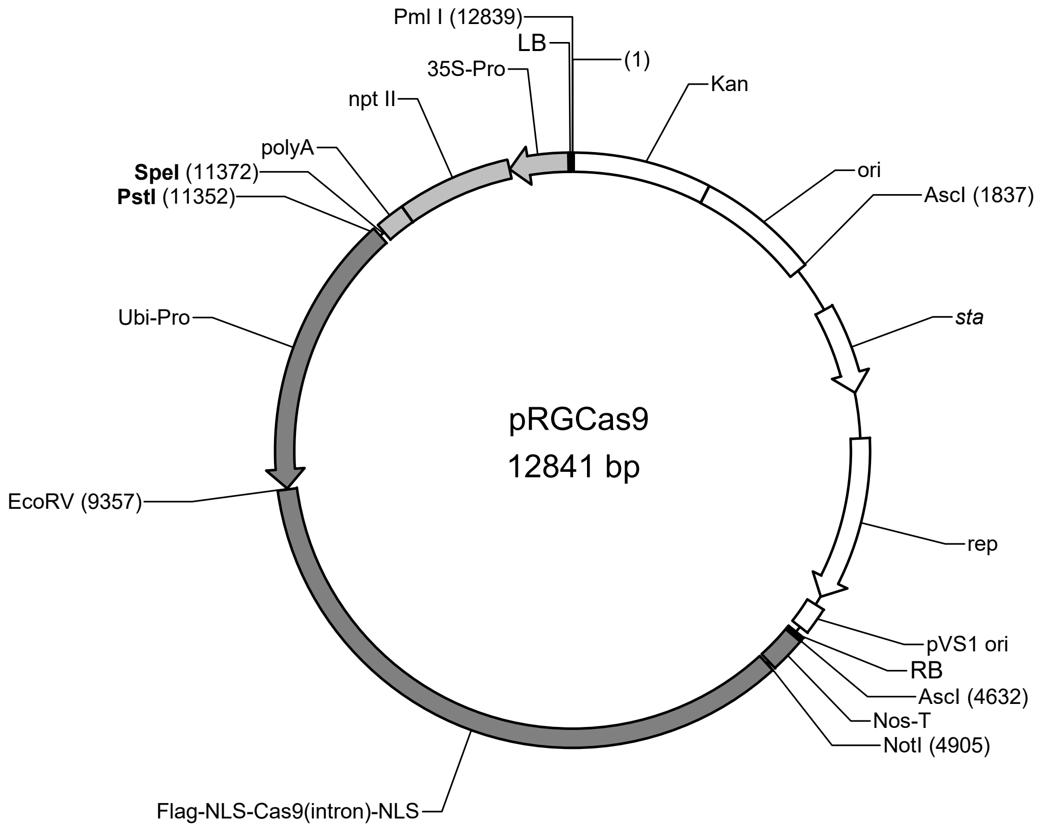
Pml I (12839)
LB
(1)
35S-Pro
Kan
npt II
polyA
SpeI (11372)
PstI (11352)
ori
AscI (1837)
Ubi-Pro
sta
pRGCas9
12841 bp
EcoRV (9357)
rep
pVS1 ori
RB
AscI (4632)
Nos-T
NotI (4905)
Flag-NLS-Cas9(intron)-NLS

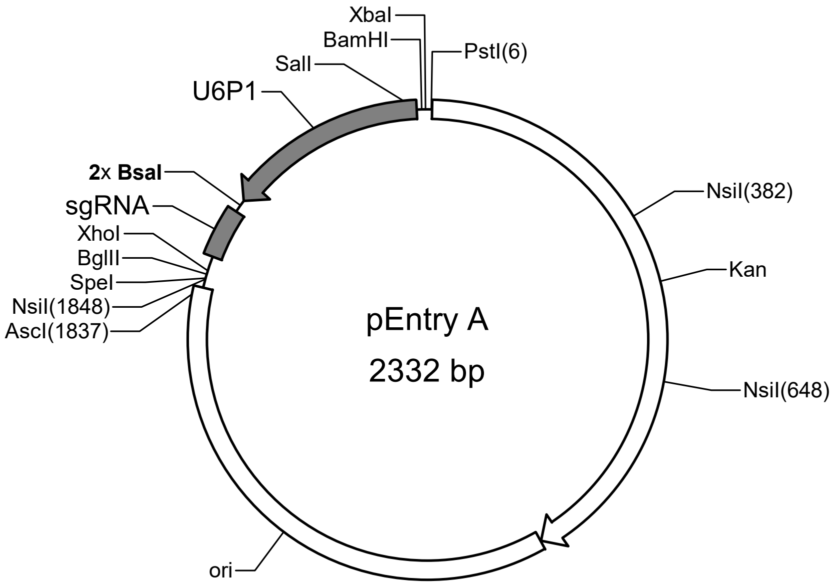
XbaI
BamHI
PstI(6)
SalI
U6P1
2x BsaI
sgRNA
XhoI
BglII
SpeI
NsiI(1848)
AscI(1837)
NsiI(382)
Kan
NsiI(648)
ori
pEntry A
2332 bp

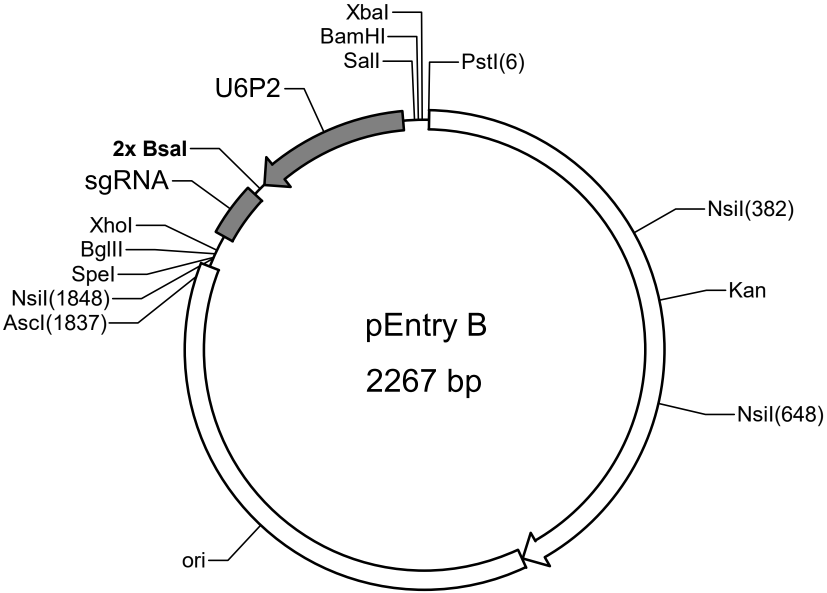
XbaI
BamHI
SalI
PstI(6)
U6P2
2x BsaI
sgRNA
XhoI
BglII
SpeI
NsiI(1848)
AscI(1837)
NsiI(382)
Kan
NsiI(648)
pEntry B
2267 bp
ori

Figure S3. The sequence structures in the MCS region of the vectors

The features of upstream and downstream sequences in the MCS region, open reading frames, restriction sites and sequencing primers included.

pRTE

```
-------------------------------------Kan complement ----------------------------  PstI   EcoRV              BamHI
TTTATGTAAGCAGACAGTTTTATTGTTCATGATGATATATTTTTATCTTGTGCAATGTAACATCAGAGATTTTGAGACAC CTGCAG GATATC CAGATCCAGTG GGA TCC CCG

SmaI  SacI    KpnI  HindIII   SpeI      NotI   3 Stop     -----------NosT-----------------------------------------
GGT GAG CTC GGT ACC AAG CTT ACT AGT GCG GCC GCt aaGtgaGtagATCGTTCAAACATTTGGCAATAAAGTTTCTTAAGATTGAATCCTGTTGCCGGTCT
```

Sequencing primer: SeqbfkanR GTAAGCAGACAGTTTTATTGTTCAT; NosR-seq AGACCGGCAACAGGATTCAATC

pRTV/pRHV/pRGV

```
--------------------------------------Ubi-Pro---------------------------------------------  EcoRV            BamHI
ATTTTTTTAGCCCTGCCTTCATACGCTATTTATTTGCTTGGTACTGTTTCTTTTGTCGATGCTCACCCTGTTGTTTGGTGTTACTTATGCAG GATATC CAGATCCAGTG GGA

    SmaI     SacI    KpnI  HindIII  SpeI     NotI   3 Stop     -----------NosT-----------------------------------
TCC CCG GGT GAG CTC GGT ACC AAG CTT ACT AGT GCG GCC GCt aaGtgaGtagATCGTTCAAACATTTGGCAATAAAGTTTCTTAAGATTGAATCCTGTTGCCGGTCT
```

Sequencing primer: UbiP-seq TTTTAGCCCTGCCTTCATACGC; NosR-seq AGACCGGCAACAGGATTCAATC

pRHE/pRGE

```
--------------------------------------------------------polyA--------------------------------------------------
TGTGAGTAGTTCCCAGATAAGGGAATTAGGGTTCCTATAGGGTTTCGCTCATGTGTTGAGCATATAAGAAACCCTTAGTATGTATTTGTATTTGTAAAATACTTCTATCAATAAAA

-----------------------------------------------------  PstI   EcoRV             BamHI   SmaI      SacI    KpnI  HindIII
TTTCTAATTCCTAAAACCAAAATCCAGTACTAAAATCCAGATCCCCCGAATTA CTGCAG GATATC CAGATCCAGTG GGA TCC CCG GGT GAG CTC GGT ACC AAG CTT

  SpeI      NotI   3 Stop     -----------NosT----------------------------------------
ACT AGT GCG GCC GCt aaGtgaGtagATCGTTCAAACATTTGGCAATAAAGTTTCTTAAGATTGAATCCTGTTGCCGGTCT
```

Sequencing primer: ployA-seq TGTGAGTAGTTCCCAGATAAGG; NosR-seq AGACCGGCAACAGGATTCAATC

pRTVnHA/pRHVnHA

```
--------------------------------------Ubi-Pro---------------------------------------------  EcoRV ----------------
ATTTTTTTAGCCCTGCCTTCATACGCTATTTATTTGCTTGGTACTGTTTCTTTTGTCGATGCTCACCCTGTTGTTTGGTGTTACTTATGCAG GATATC ATG TAC CCA TAC GAT

-------------------------------------------------4×HA------------------------------------------------------------
GTT CCA GAC TAC GCT TAC CCA TAC GAC GTT CCA GAC TAC GCT TAC CCA TAC GAC GTT CCA GAC TAC GCT GGT TAC CCA TAC GAC GTT CCA

-----------  BamHI   SmaI      SacI    KpnI  HindIII   SpeI     NotI    3 Stop     ---------NosT---------------------
GAT TAC GCT  GGA TCC CCG GGT GAG CTC GGT ACC AAG CTT ACT AGT GCG GCC GCt aaGtgaGtagATCGTTCAAACATTTGGCAATAAAGTTTCTTAAG

---------------------
ATTGAATCCTGTTGCCGGTCT
```

Sequencing primer: UbiP-seq TTTTAGCCCTGCCTTCATACGC; NosR-seq AGACCGGCAACAGGATTCAATC

pRTVcHA/pRHVcHA/pRGVcHA

```
--------------------------------------Ubi-Pro---------------------------------------------  EcoRV            BamHI
ATTTTTTTAGCCCTGCCTTCATACGCTATTTATTTGCTTGGTACTGTTTCTTTTGTCGATGCTCACCCTGTTGTTTGGTGTTACTTATGCAG GATATC CAGATCCAGTG GGA

    SmaI     SacI    KpnI  HindIII  SpeI     NotI     --------------------------------4×HA------------------------
TCC CCG GGT GAG CTC GGT ACC AAG CTT ACT AGT GCG GCC GCT ATG TAC CCA TAC GAT GTT CCA GAC TAC GCT TAC CCA TAC GAC GTT CCA

----------------------------------------------------------------------------------- 4 Stop                --------
GAC TAC GCT TAC CCA TAC GAC GTT CCA GAC TAC GCT GGT TAC CCA TAC GAC GTT CCA GAT TAC GCT tga CGGCCGCtaaGtgaGtagATCGTTCAA

-----------NosT-------------------------------
ACATTTGGCAATAAAGTTTCTTAAGATTGAATCCTGTTGCCGGTCT
```

Sequencing primer: UbiP-seq TTTTAGCCCTGCCTTCATACGC; NosR-seq AGACCGGCAACAGGATTCAATC

pRHEcHA/pRGEcHA

--polyA--

TGTGAGTAGTTCCCAGATAAGGGAATTAGGGTTCCTATAGGGTTTCGCTCATGTGTTGAGCATATAAGAAACCCTTAGTATGTATTTGTATTTGTAAAATACTTCTATCAATAAAA

--- PstI EcoRV BamHI SmaI SacI KpnI HindIII

TTTCTAATTCCTAAAACCAAAATCCAGTACTAAAATCCAGATCCCCCGAATTA CTGCAG GATATC CAGATCCAGTG GGA TCC CCG GGT GAG CTC GGT ACC AAG CTT

SpeI NotI --4×HA--

ACT AGT GCG GCC GCT ATG TAC CCA TAC GAT GTT CCA GAC TAC GCT TAC CCA TAC GAC GTT CCA GAC TAC GCT TAC CCA TAC GAC GTT CCA

-- **4 Stop** --------------**NosT**--------------------------

GAC TAC GCT GGT TAC CCA TAC GAC GTT CCA GAT TAC GCT tgaCGGCCGCtaaGtgaGtagATCGTTCAAACATTTGGCAATAAAGTTTCTTAAGATTGAATCCT

GTTGCCGGTCT

Sequencing primer: ployA-seq TGTGAGTAGTTCCCAGATAAGG; NosR-seq AGACCGGCAACAGGATTCAATC

pRTVnMyc/pRHVnMyc

---------------------------------------Ubi-Pro-- EcoRV ----------------

ATTTTTTTAGCCCTGCCTTCATACGCTATTTATTTGCTTGGTACTGTTTCTTTTGTCGATGCTCACCCTGTTGTTTGGTGTTACTTATGCAG GATATC ATG GAG CAA AAG CTC

---4×cMyc---

ATT TCT GAA GAG GAC TTG AAT GAA ATG GAG CAA AAG CTC ATT TCT GAA GAG GAC TTG AAT GAA ATG GAG CAA AAG CTC ATT TCT GAA GAG

-- BamHI SmaI

GAC TTG AAT GAA ATG GAG AGC TTG GGC GAC CTC ACC ATG GAG CAA AAG CTC ATT TCT GAA GAG GAC TTG AAT TCG GGA TCC CCG GGT GAG

SacI KpnI HindIII SpeI NotI **3 Stop** ------------------------------**NosT**---------------------

CTC GGT ACC AAG CTT ACT AGT GCG GCC GCt aaGtgaGtagATCGTTCAAACATTTGGCAATAAAGTTTCTTAAGATTGAATCCTGTTGCCGGTCT

Sequencing primer: UbiP-seq TTTTAGCCCTGCCTTCATACGC; NosR-seq AGACCGGCAACAGGATTCAATC

pRTVcMyc/pRHVcMyc

---------------------------------------Ubi-Pro-- EcoRV BamHI

ATTTTTTTAGCCCTGCCTTCATACGCTATTTATTTGCTTGGTACTGTTTCTTTTGTCGATGCTCACCCTGTTGTTTGGTGTTACTTATGCAG GATATC CAGATCCAGTG GGA

SmaI SacI KpnI HindIII SpeI NotI --------------------------------3×cMyc-----------------------

TCC CCG GGT GAG CTC GGT ACC AAG CTT ACT AGT GCG GCC GCT ATG GAG CAA AAG CTC ATT TCT GAA GAG GAC TTG AAT GAA ATG GAG CAA

AAG CTC ATT TCT GAA GAG GAC TTG AAT GAA ATG GAG AGC TTG GGC GAC CTC ACC ATG GAG CAA AAG CTC ATT TCT GAA GAG GAC TTG AAT

--- **4 Stop** --------------**NosT**------------------------------------

TCG tgaCGGCCGCtaaGtgaGtagATCGTTCAAACATTTGGCAATAAAGTTTCTTAAGATTGAATCCTGTTGCCGGTCT

Sequencing primer: UbiP-seq TTTTAGCCCTGCCTTCATACGC; NosR-seq AGACCGGCAACAGGATTCAATC

pRHEcMyc

--polyA--

TGTGAGTAGTTCCCAGATAAGGGAATTAGGGTTCCTATAGGGTTTCGCTCATGTGTTGAGCATATAAGAAACCCTTAGTATGTATTTGTATTTGTAAAATACTTCTATCAATAAAA

--- PstI EcoRV BamHI SmaI SacI KpnI HindIII

TTTCTAATTCCTAAAACCAAAATCCAGTACTAAAATCCAGATCCCCCGAATTA CTGCAG GATATC CAGATCCAGTG GGA TCC CCG GGT GAG CTC GGT ACC AAG CTT

SpeI NotI --3×cMyc--

ACT AGT GCG GCC GCT ATG GAG CAA AAG CTC ATT TCT GAA GAG GAC TTG AAT GAA ATG GAG CAA AAG CTC ATT TCT GAA GAG GAC TTG AAT

--- **4 Stop** ----**NosT**-----

GAA ATG GAG AGC TTG GGC GAC CTC ACC ATG GAG CAA AAG CTC ATT TCT GAA GAG GAC TTG AAT TCG tgaCGGCCGCtaaGtgaGtagATCGTTCAAACATT

TGGCAATAAAGTTTCTTAAGATTGAATCCTGTTGCCGGTCT

Sequencing primer: ployA-seq TGTGAGTAGTTCCCAGATAAGG; NosR-seq AGACCGGCAACAGGATTCAATC

pRGEcMyc

```
---------------------------------------------------------polyA----------------------------------------------------
TGTGAGTAGTTCCCAGATAAGGGAATTAGGGTTCCTATAGGGTTTCGCTCATGTGTTGAGCATATAAGAAACCCTTAGTATGTATTTGTATTTGTAAAATACTTCTATCAATAAAA

------------------------------------------------------  PstI   EcoRV              BamHI   SmaI      SacI    KpnI  HindIII
TTTCTAATTCCTAAAACCAAAATCCAGTACTAAAATCCAGATCCCCCGAATTA CTGCAG GATATC CAGATCCAGTG GGA TCC CCG GGT GAG CTC GGT ACC AAG CTT

  SpeI      NotI    ------------------------------------------------------------------------------------------
ACT AGT GCG GCC GCT ATG GAG CAA AAG CTC ATT TCT GAA GAG GAC TTG AAT GAA ATG GAG CAA AAG CTC ATT TCT GAA GAG GAC TTG AAT

------------------------------------------------------- 4×cMyc ---------------------------------------------------------
GAA ATG GAG CAA AAG CTC ATT TCT GAA GAG GAC TTG AAT GAA ATG GAG AGC TTG GGC GAC CTC ACC ATG GAG CAA AAG CTC ATT TCT GAA

------------------- 4 Stop                 -------------NosT--------------------------------------
GAG GAC TTG AAT TCG tgaCGGCCGCtaaGtgaGtagATCGTTCAAACATTTGGCAATAAAGTTTCTTAAGATTGAATCCTGTTGCCGGTCT
```

Sequencing primer: ployA-seq TGTGAGTAGTTCCCAGATAAGG; NosR-seq AGACCGGCAACAGGATTCAATC

pRGVcMyc

```
---------------------------------------Ubi-Pro------------------------------------------------  EcoRV              BamHI
ATTTTTTTAGCCCTGCCTTCATACGCTATTTATTTGCTTGGTACTGTTTCTTTTGTCGATGCTCACCCTGTTGTTTGGTGTTACTTATGCAG GATATC CAGATCCAGTG GGA 

    SmaI      SacI    KpnI  HindIII  SpeI     NotI     -------------------------------------------------------------
TCC CCG GGT GAG CTC GGT ACC AAG CTT ACT AGT GCG GCC GCT ATG GAG CAA AAG CTC ATT TCT GAA GAG GAC TTG AAT GAA ATG GAG CAA

---------------------------------------4×cMyc------------------------------------------------------------------------
AAG CTC ATT TCT GAA GAG GAC TTG AAT GAA ATG GAG CAA AAG CTC ATT TCT GAA GAG GAC TTG AAT GAA ATG GAG AGC TTG GGC GAC CTC

------------------------------------------------------- 4 Stop                -------------NosT-----------------------
ACC ATG GAG CAA AAG CTC ATT TCT GAA GAG GAC TTG AAT TCG tgaCGGCCGCtaaGtgaGtagATCGTTCAAACATTTGGCAATAAAGTTTCTTAAGATTGAA

---------------
TCCTGTTGCCGGTCT
```

Sequencing primer: UbiP-seq TTTTAGCCCTGCCTTCATACGC; NosR-seq AGACCGGCAACAGGATTCAATC

pRTVnGFP/pRHVnGFP

```
----------------------Ubi-Pro---------  EcoRV -------------------------------------EGFP------------------------------
GTCGATGCTCACCCTGTTGTTTGGTGTTACTTATGCAG GATATC ATG GTG AGC AAG GGC GAG GAG CTG TTC ACC---AAG CGC GAT CAC ATG GTC CTG CTG

------------------------------------------------------------------  BamHI  SmaI      SacI    KpnI  HindIII  SpeI
GAG TTC GTG ACC GCC GCC GGG ATC ACT CTC GGC ATG GAC GAG CTG TAC AAG GGA TCC CCG GGT GAG CTC GGT ACC AAG CTT ACT AGT GCG

NotI   3 Stop       -------------------------NosT-------------------------
GCC GCt aaGtgaGtagATCGTTCAAACATTTGGCAATAAAGTTTCTTAAGATTGAATCCTGTTGCCGGTCT
```

Sequencing primer: EGFP/Venus-Seq GCGATCACATGGTCCTGC; NosR-seq AGACCGGCAACAGGATTCAATC

pRTVcGFP/pRHVcGFP/pRGVcGFP

```
---------------------------------------Ubi-Pro------------------------------------------------  EcoRV              BamHI
ATTTTTTTAGCCCTGCCTTCATACGCTATTTATTTGCTTGGTACTGTTTCTTTTGTCGATGCTCACCCTGTTGTTTGGTGTTACTTATGCAG GATATC CAGATCCAGTG GGA 

    SmaI      SacI    KpnI  HindIII  SpeI     NotI     -------------------------------------------------------------
TCC CCG GGT GAG CTC GGT ACC AAG CTT ACT AGT GCG GCC GCT ATG GTG AGC AAG GGC GAG GAG CTG TTC ACC GGG GTG GTG CCC ATC CTG

---------------------------------------------------- EGFP --------------------------------  4 Stop               -NosT-
GTC GAG CTG GAC GGC GAC GTA AAC GGC CAC AAG TTC AGC---ACT CTC GGC ATG GAC GAG CTG TAC AAG taaCGGCCGCtaaGtgaGtagATCGT
```

Sequencing primer: UbiP-seq TTTTAGCCCTGCCTTCATACGC; Venus/GFP/CFPseqR AACTTGTGGCCGTTTACGTCG

pRTEcYFP

```
--------------------------------------Kan complement ----------------------------  PstI   EcoRV               BamHI
TTTATGTAAGCAGACAGTTTTATTGTTCATGATGATATATTTTTATCTTGTGCAATGTAACATCAGAGATTTTGAGACAC CTGCAG GATATC CAGATCCAGTG GGA TCC CCG

SmaI  SacI    KpnI   HindIII   SpeI      NotI    ----------------------------mVenus-------------------------------------
GGT GAG CTC GGT ACC AAG CTT ACT AGT GCG GCC GCT ATG GTG AGC AAG GGC GAG GAG CTG TTC ACC GGG GTG GTG CCC ATC CTG GTC GAG

--------------------------------------------------------------------
CTG GAC GGC GAC GTA AAC GGC CAC AAG TTC AGC---GGC ATG GAC GAG CTG TAC AAG TAACGGCCGCtaaGtgaGtagATCGTTCAAACATTTGGCAATAAAGTT
```

Sequencing primer: SeqbfkanR GTAAGCAGACAGTTTTATTGTTCAT; Venus/GFP/CFPseqR AACTTGTGGCCGTTTACGTCG

pRTVnYFP

```
----------------------Ubi-Pro---------  EcoRV -------------------------------------mVenus----------------------------
GTCGATGCTCACCCTGTTGTTTGGTGTTACTTATGCAG GATATC ATG GTG AGC AAG GGC GAG GAG CTG TTC ACC---AAG CGC GAT CAC ATG GTC CTG CTG

-------------------------------------------------------------------  BamHI  SmaI      SacI    KpnI   HindIII  SpeI
GAG TTC GTG ACC GCC GCC GGG ATC ACT CTC GGC ATG GAC GAG CTG TAC AAG GGA TCC CCG GGT GAG CTC GGT ACC AAG CTT ACT AGT GCG

NotI  3 Stop      -------------------------NosT--------------------------
GCC GCt aaGtgaGtagATCGTTCAAACATTTGGCAATAAAGTTTCTTAAGATTGAATCCTGTTGCCGGTCT
```

Sequencing primer: EGFP/Venus-Seq GCGATCACATGGTCCTGC; NosR-seq AGACCGGCAACAGGATTCAATC

pRTVcYFP

```
--------------------------------------Ubi-Pro-----------------------------------------------  EcoRV              BamHI
ATTTTTTTAGCCCTGCCTTCATACGCTATTTATTTGCTTGGTACTGTTTCTTTTGTCGATGCTCACCCTGTTGTTTGGTGTTACTTATGCAG GATATC CAGATCCAGTG GGA

   SmaI     SacI     KpnI   HindIII   SpeI     NotI     ----------------------------------------------------------------
TCC CCG GGT GAG CTC GGT ACC AAG CTT ACT AGT GCG GCC GCT ATG GTG AGC AAG GGC GAG GAG CTG TTC ACC GGG GTG GTG CCC ATC CTG

---------------------------------------------------- mVenus --------------------------------  4 Stop              -NosT-
GTC GAG CTG GAC GGC GAC GTA AAC GGC CAC AAG TTC AGC---ACT CTC GGC ATG GAC GAG CTG TAC AAG taaCGGCCGCtaaGtgaGtagATCGT
```

Sequencing primer: UbiP-seq TTTTAGCCCTGCCTTCATACGC; Venus/GFP/CFPseqR AACTTGTGGCCGTTTACGTCG

pRHEcYFP/pRGEcYFP

```
---------------------------------------------------------polyA----------------------------------------------------------
TGTGAGTAGTTCCCAGATAAGGGAATTAGGGTTCCTATAGGGTTTCGCTCATGTGTTGAGCATATAAGAAACCCTTAGTATGTATTTGTATTTGTAAAATACTTCTATCAATAAAA

------------------------------------------------------  PstI   EcoRV                BamHI   SmaI      SacI    KpnI  HindIII
TTTCTAATTCCTAAAACCAAAATCCAGTACTAAAATCCAGATCCCCCGAATTA CTGCAG GATATC CAGATCCAGTG GGA TCC CCG GGT GAG CTC GGT ACC AAG CTT

SpeI     NotI     ---------------------------------------------------------------- mVenus ----------------------------
ACT AGT GCG GCC GCT ATG GTG AGC AAG GGC GAG GAG CTG TTC ACC GGG GTG GTG CCC ATC CTG GTC GAG CTG GAC GGC GAC GTA AAC GGC

------------------------------------------------------  4 Stop              -NosT-
CAC AAG TTC AGC---ACT CTC GGC ATG GAC GAG CTG TAC AAG taaCGGCCGCtaaGtgaGtagATCGT
```

Sequencing primer: ployA-seq TGTGAGTAGTTCCCAGATAAGG; Venus/GFP/CFPseqR AACTTGTGGCCGTTTACGTCG

pRTVnRFP

```
----------------------Ubi-Pro---------  EcoRV -------------------------------------TagRFP----------------------------
GTCGATGCTCACCCTGTTGTTTGGTGTTACTTATGCAG GATATC ATG AGC GAG CTG ATT AAG GAG AAC ATG CAC---AAA GAG ACC TAC GTC GAG CAG CAC

-------------------------------------------------------------------  BamHI  SmaI      SacI    KpnI   HindIII  SpeI
GAG GTG GCT GTG GCC AGA TAC TGC GAC CTC CCT AGC AAA CTG GGG CAC AAGGGA TCC CCG GGT GAG CTC GGT ACC AAG CTT ACT AGT GCG

NotI  3 Stop       -------------------------NosT--------------------------
GCC GCt aaGtgaGtagATCGTTCAAACATTTGGCAATAAAGTTTCTTAAGATTGAATCCTGTTGCCGGTCT
```

Sequencing primer: tRFP-Seq AGAGACCTACGTCGAGCAGC ; NosR-seq AGACCGGCAACAGGATTCAATC

pRTVcRFP/pRHVcRFP/pRGVcRFP

```
--------------------------------------Ubi-Pro-----------------------------------------------  EcoRV             BamHI
ATTTTTTTAGCCCTGCCTTCATACGCTATTTATTTGCTTGGTACTGTTTCTTTTGTCGATGCTCACCCTGTTGTTTGGTGTTACTTATGCAG  GATATC  CAGATCCAGTG  GGA

    SmaI      SacI     KpnI   HindIII   SpeI      NotI      -------------------------------------------------------------
TCC CCG GGT  GAG CTC  GGT ACC  AAG CTT  ACT AGT  GCG GCC GCT  ATG AGC GAG CTG ATT AAG GAG AAC ATG CAC ATG AAG CTG TAC ATG GAG

-------------------------------- TagRFP -------------------------------  4 Stop                 -NosT-
GGC ACC GTG AAC AAC CAC CAC TTC AAG--- GAC CTC CCT AGC AAA CTG GGG CAC AAG  tgaCGGCCGCtaaGtgaGtagATCGT
```

Sequencing primer: UbiP-seq TTTTAGCCCTGCCTTCATACGC; tRFPseqR AAGTGGTGGTTGTTCACGGTGC

pRTVnVN

```
--------------------------------------Ubi-Pro-----------------------------------------------  EcoRV  -----------------
ATTTTTTTAGCCCTGCCTTCATACGCTATTTATTTGCTTGGTACTGTTTCTTTTGTCGATGCTCACCCTGTTGTTTGGTGTTACTTATGCAG GATATC ATG GTG AGC AAG GGC

--------------------mVenus N(1-155)----------------------  ----------------HA----------------   BamHI  SmaI     SacI
GAG GAG CTG TTC ACC---AGC CAC AAC GCC TAT CTC ACC GCC GAC  TAC CCA TAC GAT GTT CCA GAT TAC GCT  GGA TCC CCG GGT  GAG CTC

 KpnI   HindIII   SpeI     NotI   3 Stop    -------------------------NosT--------------------------
GGT ACC  AAG CTT  ACT AGT  GCG GCC GCt  aaGtgaGtagATCGTTCAAACATTTGGCAATAAAGTTTCTTAAGATTGAATCCTGTTGCCGGTCT
```

Sequencing primer: UbiP-seq TTTTAGCCCTGCCTTCATACGC; NosR-seq AGACCGGCAACAGGATTCAATC

pRTVnVC

```
----------------------Ubi-Pro---------  EcoRV  -------------------------------------mVenus C(156-238)-----------------
GTCGATGCTCACCCTGTTGTTTGGTGTTACTTATGCAG  GATATC ATG AAG CAG AAG AAC GGC ATC AAG GCC AAC---GTC CTG CTG GAG TTC GTG ACC  GCC

----------------------------------------------  ---------------------cMyc--------------------   BamHI   SmaI     SacI
GCC GGG ATC ACT CTC GGC ATG GAC GAG CTG TAC AAG  GAG CAA AAG CTC ATT TCT GAA GAG GAC TTG AAT GAA  GGA TCC CCG GGT  GAG CTC

 KpnI   HindIII   SpeI      NotI   3 Stop    -------------------------NosT--------------------------
GGT ACC  AAG CTT  ACT AGT  GCG GCC GCt  aaGtgaGtagATCGTTCAAACATTTGGCAATAAAGTTTCTTAAGATTGAATCCTGTTGCCGGTCT
```

Sequencing primer: VC-Seq TCCTGCTGGAGTTCGTGACC; NosR-seq AGACCGGCAACAGGATTCAATC

pRTVcVN

```
--------------------------------------Ubi-Pro-----------------------------------------------  EcoRV             BamHI
ATTTTTTTAGCCCTGCCTTCATACGCTATTTATTTGCTTGGTACTGTTTCTTTTGTCGATGCTCACCCTGTTGTTTGGTGTTACTTATGCAG  GATATC  CAGATCCAGTG  GGA

    SmaI      SacI     KpnI   HindIII   SpeI      NotI       ----------------HA---------------  --------------------------
TCC CCG GGT  GAG CTC  GGT ACC  AAG CTT  ACT AGT  GCG GCC GCT  TAC CCA TAC GAT GTT CCA GAT TAC GCT  GTG AGC AAG GGC GAG GAG CTG

----------- mVenus N(1-155) --------------------  4 Stop                ----------------------NosT---------------------
TTC ACC---AAC AGC CAC AAC GCC TAT CTC ACC GCC GAC  taaCGGCCGCtaaGtgaGtagATCGTTCAAACATTTGGCAATAAAGTTTCTTAAGATTGAATCCTGTT

--------
GCCGGTCT
```

Sequencing primer: UbiP-seq TTTTAGCCCTGCCTTCATACGC; NosR-seq AGACCGGCAACAGGATTCAATC

pRTVcVC

```
--------------------------------------Ubi-Pro-----------------------------------------------  EcoRV             BamHI
ATTTTTTTAGCCCTGCCTTCATACGCTATTTATTTGCTTGGTACTGTTTCTTTTGTCGATGCTCACCCTGTTGTTTGGTGTTACTTATGCAG  GATATC  CAGATCCAGTG  GGA

    SmaI      SacI     KpnI   HindIII   SpeI      NotI       -------------------- cMyc --------------------  ---------------
TCC CCG GGT  GAG CTC  GGT ACC  AAG CTT  ACT AGT  GCG GCC GCT  GAG CAA AAG CTC ATT TCT GAA GAG GAC TTG AAT GAA  AAG CAG AAG AAC

--------------------------------------mVenus C(156-238)----------------------------------  4 Stop               -NosT-
GGC ATC AAG GCC AAC TTC AAG ATC CGC CAC AAC ATC GAG GAC---ATC ACT CTC GGC ATG GAC GAG CTG TAC AAG  taaCGGCCGCtaaGtgaGtagATCGT
```

Sequencing primer: UbiP-seq TTTTAGCCCTGCCTTCATACGC; VC seqR CGATGTTGTGGCGGATCTTGA

pEntry A

-------------------------------------**Kan complement** ---------------------------- PstI XbaI BamHI SalI

TTTATGTAAGCAGACAGTTTTATTGTTCATGATGATATATTTTTATCTTGTGCAATGTAACATCAGAGATTTTGAGACAC CTGCAG TCTAGA GGATCC GTCGAC

----------------**U6P1**-------------------- **2×BsaI** ------**sgRNA**----------- XhoI BglII SpeI NsiI

AAGAACGAACTAAGCCGGAC---GCGCTGCCGCTTG**TGTT**AgagaccAAAGGAggtctcA**GTTT**TAGAGCTAGAAATAG---CTCGAG AGATCT ACTAGT ATGCAT--

Sequencing primer: SeqbfkanR GTAAGCAGACAGTTTTATTGTTCAT

pEntry B

-------------------------------------**Kan complement** ---------------------------- PstI XbaI BamHI SalI

TTTATGTAAGCAGACAGTTTTATTGTTCATGATGATATATTTTTATCTTGTGCAATGTAACATCAGAGATTTTGAGACAC CTGCAG TCTAGA GGATCC GTCGAC

----------------**U6P2**--------------------- **2×BsaI** ------**sgRNA**----------- XhoI BglII SpeI NsiI

TGCTGGAATTGCCCTTGGAT---CGCGCTGTCGCTT**GTGT**AgagaccAAAGGAggtctcA**GTTT**TAGAGCTAGAAATAG---CTCGAG AGATCT ACTAGT ATGCAT--

Sequencing primer: SeqbfkanR GTAAGCAGACAGTTTTATTGTTCAT

pRHCas9/pRGCas9

-----------------------------------**Ubi-Pro complement** -------------------------- PstI ~~BamHI~~

AAAATATGTGGTAATTTTTTATAACTTAGACATGCAATGCTCATTATCTCTAGAGAGGGGCACGACCGGGTCACGCTGCA CTGCAG ACGTGA GGATCC ACTGAG

SpeI ------------**polyA Complement**-----------

ACTAGT ATGCAG TAATTCGGGGGATCTGGATTTTAGTACTGGATTTTGGTT

Sequencing primer: SeqbfUbiR GGTAATTTTTTATAACTTAGACATGC

Table S1. List of the 42 vectors generated in this study

No.	Vector name	Description
1	pRTE	Transient expression, without Ubi promoter, tag free
2	pRTEcYFP	Transient expression, without Ubi promoter, C terminal mVenus tag
3	pRTV	Transient over-expression, Ubi promoter, tag free
4	pRTVnHA	Transient over-expression, Ubi promoter, N terminal 4×HA tag
5	pRTVnMyc	Transient over-expression, Ubi promoter, N terminal 4×cMyc tag
6	pRTVnGFP	Transient over-expression, Ubi promoter, N terminal EGFP tag
7	pRTVnYFP	Transient over-expression, Ubi promoter, N terminal mVenus tag
8	pRTVnRFP	Transient over-expression, Ubi promoter, N terminal TagRFP tag
9	pRTVcHA	Transient over-expression, Ubi promoter, C terminal 4×HA tag
10	pRTVcMyc	Transient over-expression, Ubi promoter, C terminal 3×cMyc tag
11	pRTVcGFP	Transient over-expression, Ubi promoter, C terminal EGFP tag
12	pRTVcYFP	Transient over-expression, Ubi promoter, C terminal mVenus tag
13	pRTVcRFP	Transient over-expression, Ubi promoter, C terminal TagRFP tag
14	pRTVnVN	Transient BiFC, N terminal mVenusN-HA tag
15	pRTVnVC	Transient BiFC, N terminal mVenusC-cMyc tag
16	pRTVcVN	Transient BiFC, C terminal HA-mVenusN tag
17	pRTVcVC	Transient BiFC, C terminal cMyc-mVenusC tag
18	pRHE	Stable expression, Hygromycin, without Ubi promoter, tag free
19	pRHEcHA	Stable expression, Hygromycin, without Ubi promoter, C terminal 4×HA tag
20	pRHEcMyc	Stable expression, Hygromycin, without Ubi promoter, C terminal 3×cMyc tag
21	pRHEcYFP	Stable expression, Hygromycin, without Ubi promoter, C terminal mVenus tag
22	pRHV	Stable over-expression, Hygromycin, Ubi promoter, tag free
23	pRHVnHA	Stable over-expression, Hygromycin, Ubi promoter, N terminal 4×HA tag
24	pRHVnMyc	Stable over-expression, Hygromycin, Ubi promoter, N terminal 4×cMyc tag
25	pRHVnGFP	Stable over-expression, Hygromycin, Ubi promoter, N terminal EGFP tag
26	pRHVcHA	Stable over-expression, Hygromycin, Ubi promoter, C terminal 4×HA tag
27	pRHVcMyc	Stable over-expression, Hygromycin, Ubi promoter, C terminal 3×cMyc tag
28	pRHVcGFP	Stable over-expression, Hygromycin, Ubi promoter, C terminal EGFP tag
29	pRHVcRFP	Stable over-expression, Hygromycin, Ubi promoter, C terminal TagRFP tag
30	pRGE	Stable expression, G418, without Ubi promoter, tag free
31	pRGEcHA	Stable expression, G418, without Ubi promoter, C terminal 4×HA tag
32	pRGEcMyc	Stable expression, G418, without Ubi promoter, C terminal 4×cMyc tag
33	pRGEcYFP	Stable expression, G418, without Ubi promoter, C terminal mVenus tag
34	pRGV	Stable over-expression, G418, Ubi promoter, tag free
35	pRGVcHA	Stable over-expression, G418, Ubi promoter, C terminal 4×HA tag
36	pRGVcMyc	Stable over-expression, G418, Ubi promoter, C terminal 4×cMyc tag
37	pRGVcGFP	Stable over-expression, G418, Ubi promoter, C terminal EGFP tag
38	pRGVcRFP	Stable over-expression, G418, Ubi promoter, C terminal TagRFP tag
39	pRHCas9	Stable genome editing, Hygromycin, Ubi promoter, 3×Flag-NLS-Cas9-NLS
40	pRGCas9	Stable genome editing, G418, Ubi promoter, 3×Flag-NLS-Cas9-NLS
41	pEntry A	Entry vector for construction U6P1-sgRNA scaffold
42	pEntry B	Entry vector for construction U6P2-sgRNA scaffold

Table S2. List of all primers used in this paper

Primer name	Sequence	RE site	Featrues
P1	ATAT CTGCAG GTGTCTCAAAATCTCTGATGTTAC	PstI	for kan and puc ori, F
P2-3	ATAT GGCGCGCC GGGCGCTCTTCCGCTTCCT	AscI	for kan and puc ori, R
P3	CG GGATCCCCGGGT GAGCTC GGTACC AAGCTT ACTAGT GCGGCCGCT TAAGATCGTTCAAACATTTGGC	MCS	for MCS and NOST, F
P3+	ACTG GCG GCC GCT AAG TGA GTA GATCGTTCAAACATTTGGC	NotI	for 3 Stop codon
P4-3	ACTG GGCGCGCC CCGATCTAGTAACATA	AscI	for MCS and NOST, R
P5	ACTG GATATC ATG TAC CCA TAC GAT GTT	EcoRV	for multiple HA tag, F
P6	ATCG GGATCC AGCGTAATCTGGAAC	BamHI	for multiple HA tag, R
P7	ACTG GATATC ATG GAG CAA AAG CTC	EcoRV	for multiple cMyc tag, F
P8	ATCG GGATCC CGAATTCAAGTCCTC	BamHI	for multiple cMyc tag, R
P9	ACTG GATATC ATGGTGAGCAAGGGCGA	EcoRV	for multi-fluorescence.except tagRFP, F
P10	ATCG GGATCC CTTGTACAGCTCGTCCATGC	BamHI	for multi-fluorescence.except tagRFP, R
P11	CCA ATGCAT GATATC ATGAGCGAGCTGATTAAG	NsiI,EcoRV	for tagRFP, F
P12	ATCG GGATCC CTTGTGCCCCAGTTTGCTA	BamHI	for tagRFP, R
P13	CG GGATCC AGCGTAATCTGGAACATCGTATGGGTA GTCGGCGGTGAGATAGGCGTTG	BamHI	mVenusN155-HA R
P14-2	ACTG GATATC ATG AAGCAGAAGAACGGCATCA	EcoRV	for mVenusC155-F with ATG
P15	CG GGATCC TTCATTCAAGTCCTCTTCAGAAATGAGCTTTTGCTC CTTGTACAGCTCGTCCATGCCG	BamHI	mVenusC155-Myc R
P5-2	actg GCGGCCGC T ATG TAC CCA TAC GAT GTT	NotI	for HA, F, c-tag
P6-2	ACTG CGGCCG TCA AGCGTAATCTGGAACGTC	EagI	for HA, R, c-tag
P7-2	actg GCGGCCGC T ATG GAG CAA AAG CTC	NotI	for cMyc, F, c-tag
P8-2	ACTG CGGCCG TCA CGAATTCAAGTCCTC	EagI	for cMyc, R, c-tag
P9-2	actg GCGGCCGC T ATGGTGAGCAAGGGCGA	NotI	for multiflor.except tagRFP, F, c-tag
P10-2	ACTG CGGCCG TTACTTGTACAGCTCGTCCATG	EagI	for multiflor.except tagRFP, R, c-tag
P11-2	actg GCGGCCGC T ATGAGCGAGCTGATTAAG	NotI	for tagRFP, F, c-tag

Primer name	Sequence	RE site	Featrues
P12-2	ACTG CGGCCG TCACTTGTGCCCCAGTTTG	EagI	for tagRFP, R, c-tag
P9-3	actg GCGGCCGC T TACCCATACGATGTTCCAGATTACGCT GTGAGCAAGGGCGAGGAGCT	NotI	for BIFC VN, F, c-tag, HA tag
P13-2	ACTG CGGCCG TTA GTCGGCGGTGAGATAGGCGT	EagI	for BIFC VN, R, c-tag,
P14-3	actg GCGGCCGC T GAGCAAAAGCTCATTTCTGAAGAGGACTTGAATGAA AAGCAGAAGAACGGCATCA	NotI	for BIFC Vc, F, c-tag, Myc tag
P18	ACTG CTGCAG TGCAGCGTGACC	PstI	for Ubip promoter, F
P19-2	ACTG GATATC CTGCATAAGTAACACCAAACAACAGG	EcoRV	for Ubip promoter, R
P20-4	ACTG GGCGCGCC TGACAGGATATATTGGCGGGTAAAC TAGGCGCTTTTTGCAGCTCTTC	AscI	for T-DNA RB+pVS1 ori complement, F
P21MR	TCGTGGCAAGCGTCCGCTGATCGAAT		mutant NotI between pVS1 Rep and sta
P22MF	ATTCGATCAGCGGACGCTTGCCACGA		mutant NotI between pVS1 Rep and sta
P23-2	ATAT GGCGCGCC GAATGAACGCCAAGAGGAACA	AscI	for PVS1 sta, R
P24-2	CCA ATGCAT TGGCAGGATATATTGTGGTGTAAACA AACATGGTGGAGCACGACA	NsiI	for T-DNA LB and 35S, F
P24-3	CCA ATGCAT cacgtg TGGCAGGATATATTGTGGTGTA	NsiI, PmlI	for T-DNA LB and 35S, F, add PmlI
P25	TGTCCTCTCCAAATGAAATG		for 35S promoter, R
P26	GATCTGTCGATCGACAAGCT		for polyA, F
P27-2	ACTG CTGCAG TAATTCGGGGGATCTGGA	PstI	for polyA,R
P27-3	ACTG ATGCAT TAATTCGGGGGATCTGGA	NsiI	for polyA,R
P28	CATTTGGAGAGGACA ATGAAAAAGCCTGAAC		for hptII, F
P29	GATCGACAGATC CTATTTCTTTGCCCTCGGAC		for hptII, R
P32	CATTTGGAGAGGACA ATGGTGAGCAAGGGCGA		for BiFC ECFP/mCherry fusion to 35S, F
P33	GATCGACAGATC TCACTTGTACAGCTCGTCCATGCCG		for BiFC ECFP/mCherry fusion to polyA, R
P34-2	CCA ATGCAT AACATGGTGGAGCACGACA	NsiI	for BiFC 35S promoter, F
P39-2	AGTT ctgcag TCTAGA GGATCC GTCGAC AAGAACGAACTAAGCCGGAC	MCS	construct sgRNA 1 cassette, F
P40-3	TGAGACCTCCTTTGGTCTCT AACACAAGCGGCAGCGC		construct sgRNA 1 cassette, MR
P41-3	AgagaccAAAGGAggtctcAGTTTTAGAGCTAGAAATAG		construct sgRNA 1 cassette, MF

Primer name	Sequence	RE site	Featrues
P44-2	AGTT GGCGCGCC ATGCAT ACTAGT AGATCT CTCGAG AATTGCCCTTCGAAGGGAC	MCS	construct sgRNA cassette, R
P39-4	AGTT ctgcag TCTAGA GGATCC GTCGAC TGCTGGAATTGCCCTTGGATC		construct sgRNA 2 cassette, F
P45	ATC ATGGACTATAAGGATCACGATGG	1/2 EcoRV	cas9 F
P46	AGTA GCGGCCGC TCACCCGCCAACTTTCCTC	NotI	cas9 R
P47-2	CATTTGGAGAGGACA ATGGGGATTGAACAAGATGG		G418, fusion with 35S
P48	GATCGACAGATC TCAGAAGAACTCGTCAAGAAG		G418, fusion with polyA
P49	G GATATC CAGATCCAGTG G		PstI+EcoRV+BamHI, to delete Ubi-pro
P50	GATCCCACTGGATCTGGATATCCTGCA		PstI+EcoRV+BamHI, to delete Ubi-pro
P51-2	CAGGAACTTGACATTA GTAAGTTTCTGCTTCTACC		insert Intron IV2 to cas9 HNH domain
P52-2	TAATCGCTCAACCGGT CTGCACATCAACAAATTTTG		insert Intron IV2 to cas9 HNH domain
P53	GAAACTTAC TAATGTCAAGTTCCTGATCG		amplify cas9 HNH domain MR
P54	ATGTGCAG ACCGGTTGAGCGATTATGAC		amplify cas9 HNH domain MF
P55	GACGTGAGGATCCACTGAGACTAGTATGCA	MCS	Add MCS to binary vector for accepting sgRNA, F
P56	TACTAGTCTCAGTGGATCCTCACGTCTGCA	MCS	Add MCS to binary vector for accepting sgRNA, R
Adaptor F	ATCCAGATCCAGTGG		delete TagRFP in pHF215, F
Adaptor R	GATCCCACTGGATCTGGAT		delete TagRFP in pHF215, R
oriSeqF	GATACCTACAGCGTGAGCATTGAG		sequencing for ori , F
KanSeqR	CGACTGAATCCGGTGAGAATG		sequencing for kan, R
SeqbfKanR	GTAAGCAGACAGTTTTATTGTTCAT		sequencing before kan,R
SeqbfUbiR	GGTAATTTTTTATAACTTAGACATGC		sequencing before Ubi,R
IPA1-sgRNA-F	tgttGAGAGCACAGCTCGAGTCGG		CRISPR-IPA1 target, F
IPA1-sgRNA-R	aaacCCGACTCGAGCTGTGCTCTC		CRISPR-IPA1 target, R
IPA1-CSPID-F	CCAAGGGTTCCAAGCAGCGTAA		amplify CRISPR-IPA1 target region, F
IPA1-CSPID-R	TGGGCATGATGGCTAGGACCAG		amplify CRISPR-IPA1 target region, R

Primer name	Sequence	RE site	Featrues
cLUC-F	ATC ATGTCCGGTTATGTAAACAATCC	1/2 EcoRV	Construct cLUC to pRTVnMyc
cLUC-R	CACGGCGATCTTTCCGCCCTT		Construct cLUC to pRTVnMyc
LUC-F	actt GGATCC ATGGAAGACGCCAAAAACA	BamHI	Construct LUC to pRTVcVC
LUC-MR	GTTTACATAACCGGATCCATCCTTGTCAATCAAGGC		amplify LUC N terminal
LUC-MF	GATTGACAAGGATGGATCCGGTTATGTAAACAATCC		amplify LUC C terminal
LUC-R	actg AAGCTT CACGGCGATCTTTCCGCCCTT	HindIII	Construct LUC to pRTVcVC
